# Supplementary material for: Discrete vulnerability to pharmacological CDK2 inhibition is governed by heterogeneity of the cancer cell cycle
Source: Nat Commun. 2025 Feb 9;16:1476. doi: 10.1038/s41467-025-56674-4 (PMC11808123; doi:10.1038/s41467-025-56674-4)
Supplement: Supplementary file 1 — Supplementary information [file 41467_2025_56674_MOESM1_ESM.pdf]

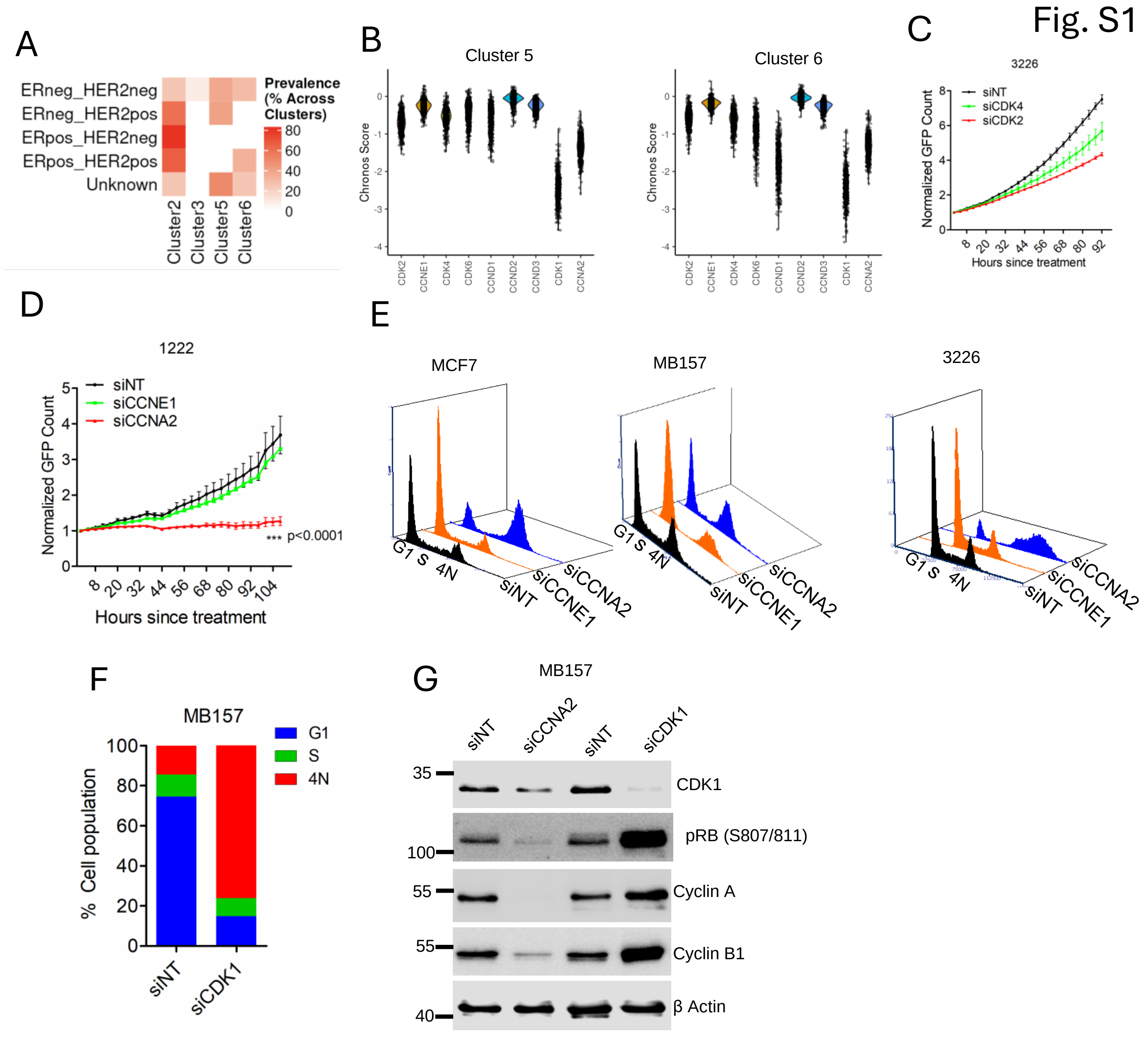

(A) Heat map depicting the enrichment of different breast cancer subtypes in each cluster. (B) Violin plots to illustrate the distribution of cell lines from cluster 5 and 6 based on their dependencies to the indicated genes. (C) Effect of CDK4 and CDK2 individual knockdowns on the proliferation of 3226 cells based on Live cell imaging. Error bars indicate mean and SD from triplicates. Experiment was done at n=3 independent times. (D) The growth of 1222 cells following the depletion of *CCNE1* and *CCNA2* using gene specific siRNAs. Error bars were calculated based on mean and SD. Experiment was done at n=3 independent times. ***p<0.0001 as determined by 2-way ANOVA. (E) Representative PI profiles from the indicated cell lines following the deletion of *CCNE1* and *CCNA2.* (F) Effect of CDK1 deletion on the cell cycle profile from MB157 cells. The column graphs represents the %population of cells at each phases of cell cycle from n=1 biological replicate. (G) Biochemical analysis to determine the differential effect of *CCNA2* and *CDK1* deletion on the indicated proteins form MB157 cells. Source data are provided in source data table.

Fig. S2

A

Effect of INX-315 based on NanoBRET assay

Complex	IC50
Cyclin E1/CDK2	2.3 nM
Cyclin A/CDK2	71 nM

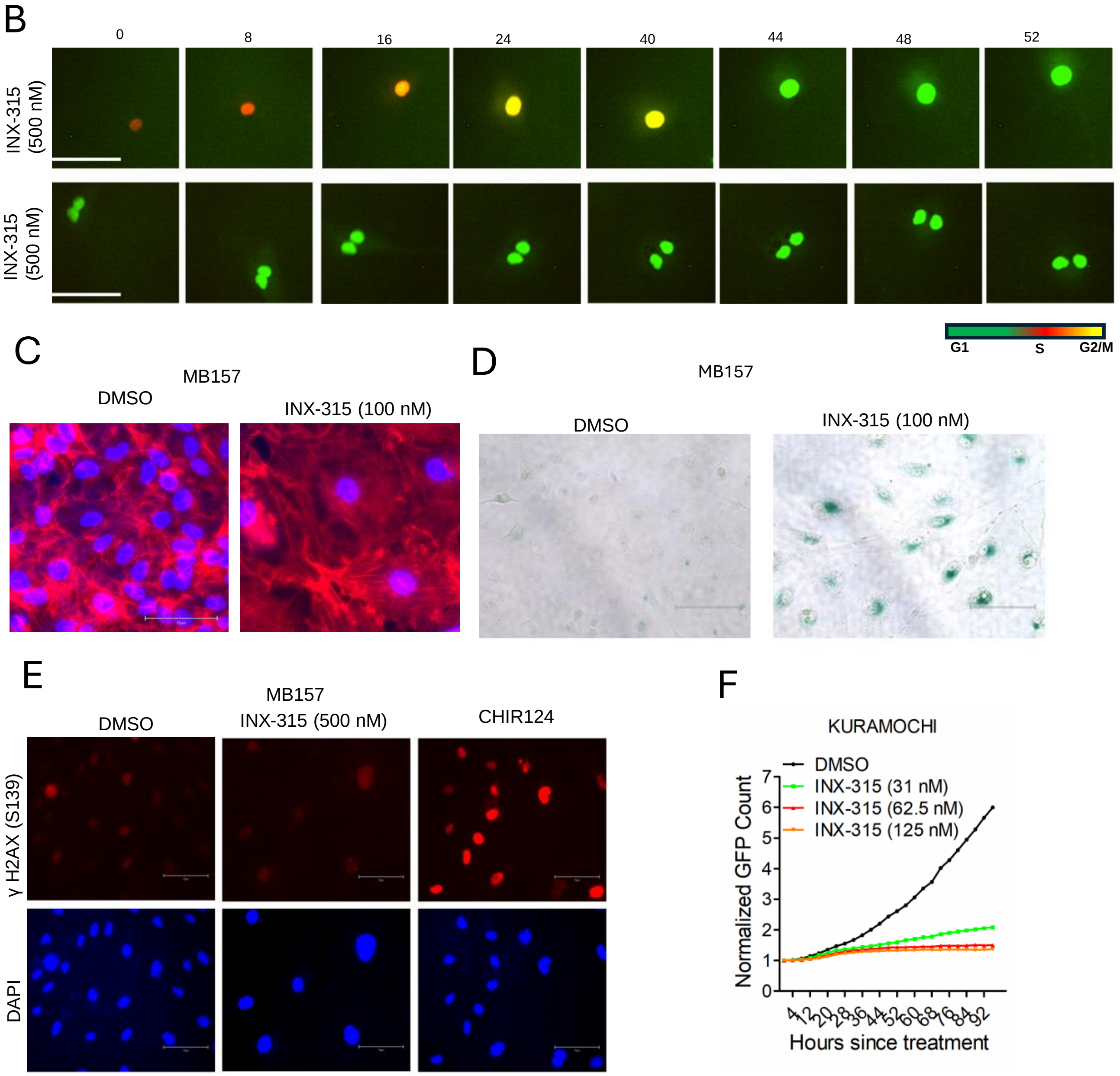

(A) NanoBRET assay quantifying INX-315's intracellular binding with cyclin E1/CDK2 and Cyclin A/CDK2 complexes. (B) Representative images of MB157 cells expressing PIP-FUCCI that were treated with INX-315 (500 nM) for the indicated time points. (C) Representative images of phalloidin staining from MB157 cells that were treated with INX-315 (100 nM) for 72 hours. Scale bar represents 75 microns. (D) Representative images of senescence-associated β-galactosidase staining in MB157 cells following the treatment with INX-315 (100 nM) for 7 days. (E) Immunofluorescence assay to determine the impact of INX-315 on DNA damage based on γH2AX staining. CHIR-124, which is a CHK1 inhibitor was used as a positive control. (F) Live cell imaging to monitor the proliferation of KURAMOCHI cell lines following the treatment with different concentrations of INX-315 until the indicated time points. Mean was calculated from n=3 technical replicates. Experiments were repeated at n=2 independent times. Source data are provided in source data table.

Fig. S3

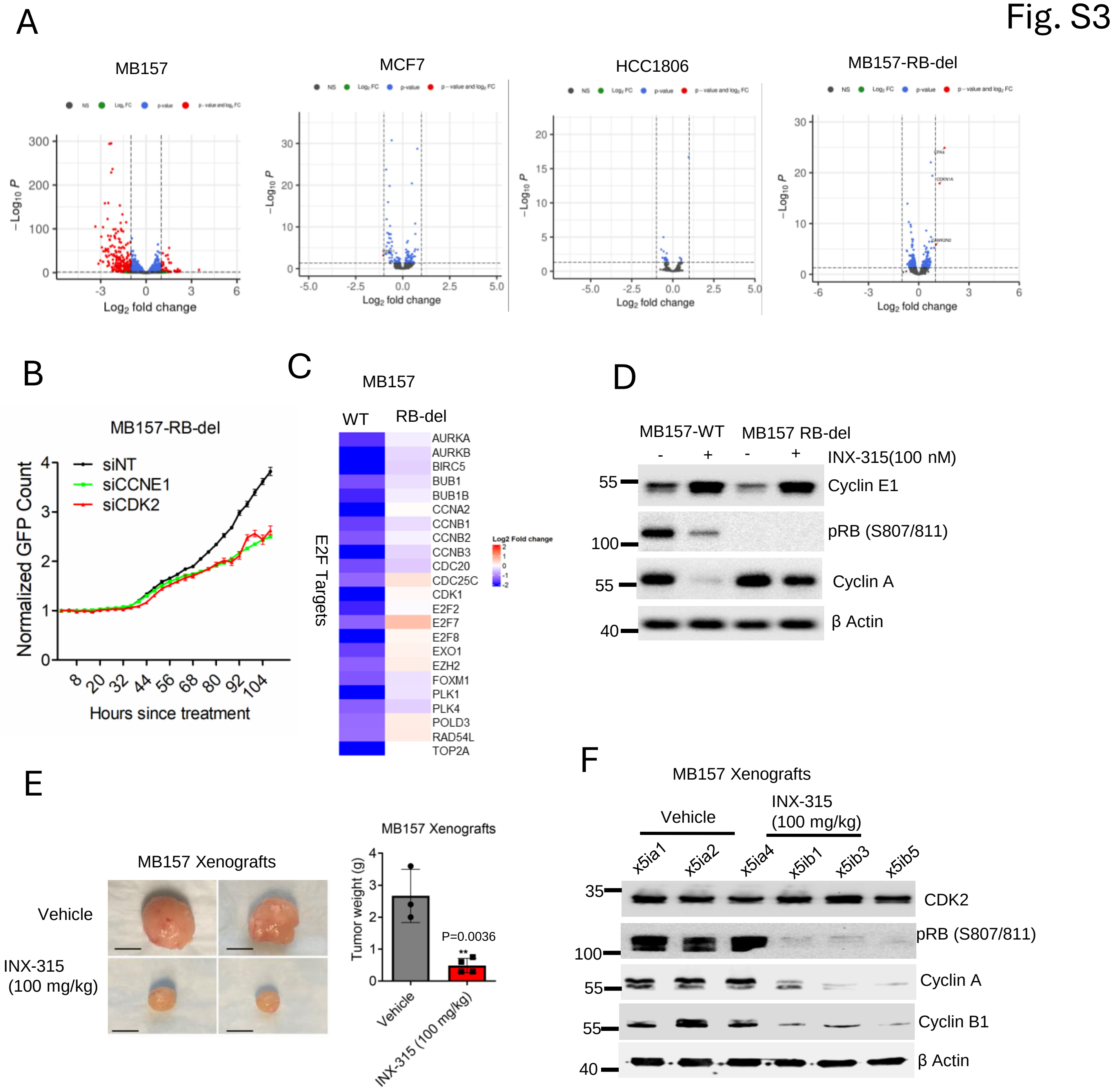

(A) Volcano plot indicating the differentially expressed genes from the indicated cell lines following the treatment with INX-315 (100 nM). (B) Live cell imaging to monitor the proliferation of MB157-RB-del cells following the depletion of *CCNE1* and *CDK2.* Error bars indicate mean and SD from triplicates. Experiment was done at n=3 independent times (C) Heatmap depicting the differential expression of the indicated E2F target genes from MB157-WT and RB-del cells following the treatment with INX-315 (100 nM). (D) Biochemical analysis comparing the effect of INX-315 on the indicated proteins from MB157-WT and RB-del cells. (E) Representative tumor images from MB157 xenografts treated with vehicle and INX-315. Column graph illustrates the tumor weights from the MB157 xenografts treated with vehicle (n=3) and INX-315 (n=4). Mean and SEM were shown. ** represents p= 0.00361 as determined by two-tailed student t-test, comparing the vehicle and INX-315-treated groups. (F) Biochemical analysis on the tumor tissues derived from mice that were treated with vehicle and INX-315 to demonstrate the impact of the drug on RB activation and cell cycle proteins. Source data are provided in source data table.

Fig. S4

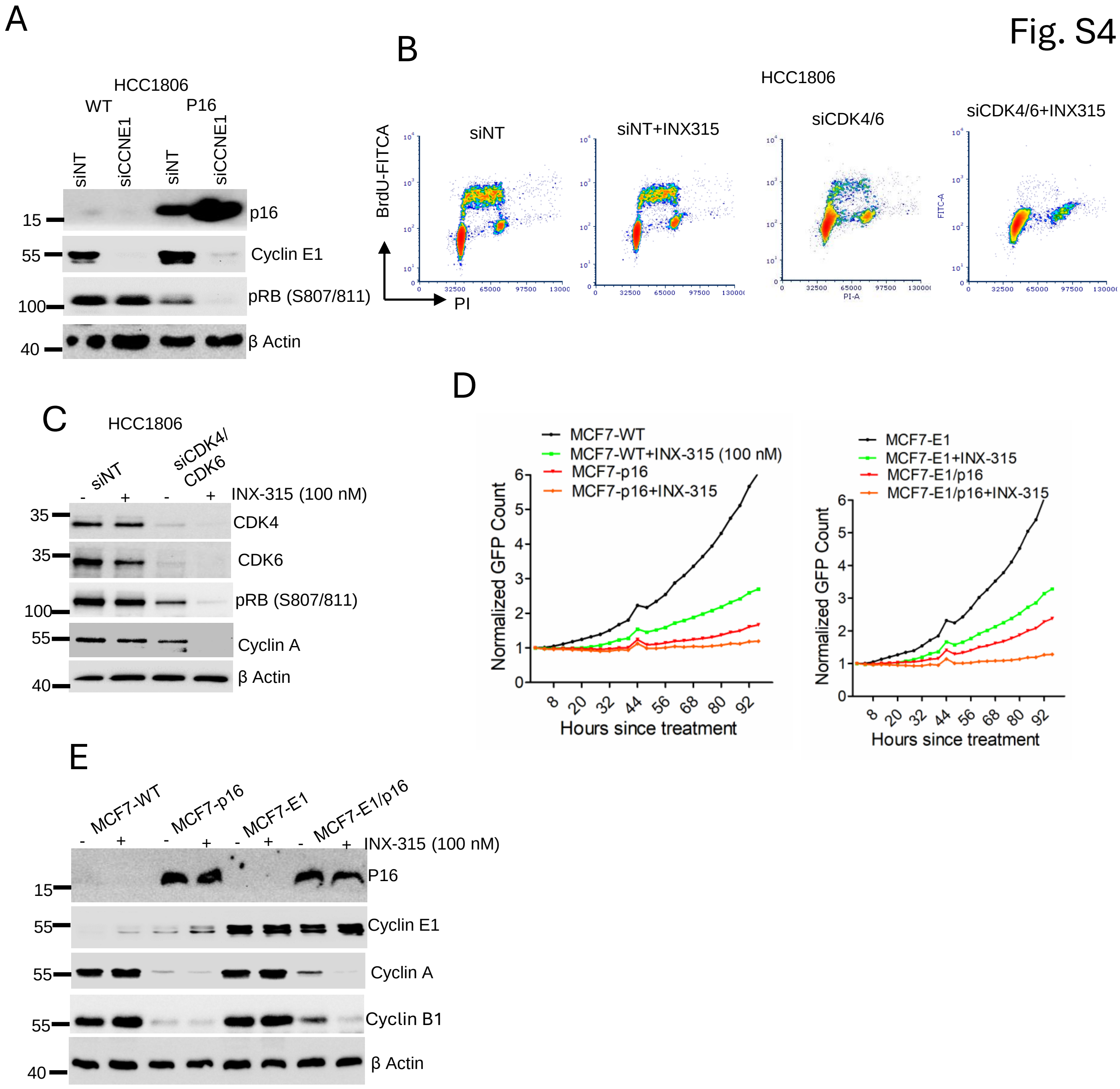

(A) Western blotting to investigate the impact of *CCNE1* deletion in HCC1806 cells following the ectopic expression of p16INK4A. (B) Bi-variate flow cytometry analysis from HCC1806 cells to elucidate the effect of concurrent deletion of CDK4 and 6 KD in combination with INX-315 on the BrdU incorporation. (C) Western blotting to evaluate the effect of concurrent deletion of CDK4 and 6 kinases on the indicated proteins in the absence and presence of INX-315 (100 nM) (D) Live cell imaging from MCF7-WT and its isogenic counterpart, MCF7-E1 cells, which harbor Tet-induced cyclin E1 overexpression. Both the cell lines were exogenously overexpressed with P16INK4A in the presence and absence of INX-315. Data points indicate mean from n=3 technical replicates. Experiment was done at two independent times. (E) Biochemical analysis on the indicated proteins following INX-315 treatment from MCF7-WT and MCF7-E1 cells that were overexpressed with P16INK4A. Source data are provided in source data table.

Fig. S5

A

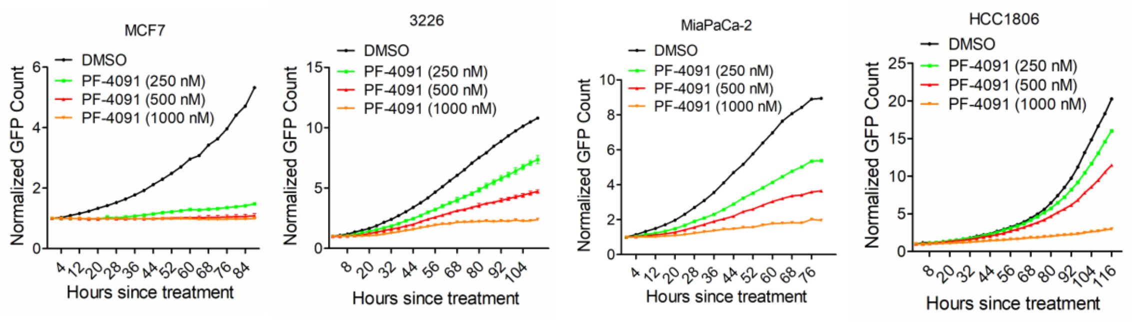

B C D

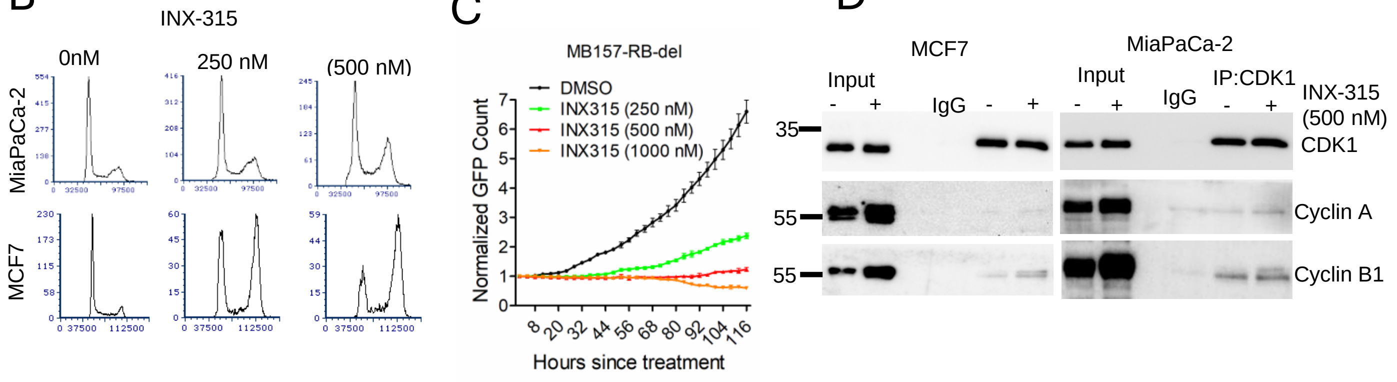

E

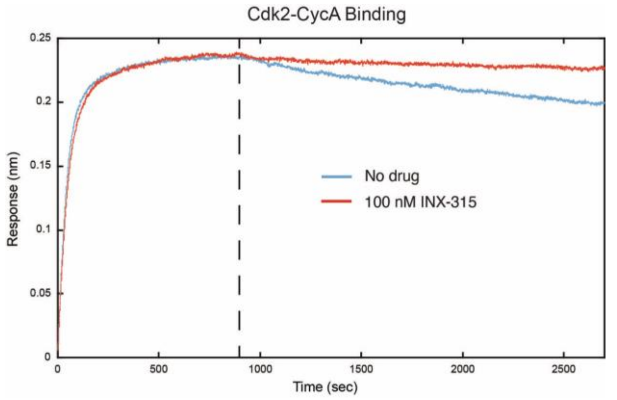

	ka (x 10^5 $M^{-1}s^{-1}$)	kd (x 10^{-4} s)	Kd (nM)
No drug	2.0 ± 0.1	1.1 ± 0.1	0.57 ± 0.04
with INX-315	1.7 ± 0.1	0.5 ± 0.1*	0.24 ± 0.03*

* $p < 0.01$, comparison to no drug

F

Effect of INX-315 based on NanoBRET assay

Complex	IC50
Cyclin B/CDK1	374 nM
Cyclin A/CDK1	536 nM

(A). Effect of PF-4091 on the proliferation of indicated cell lines following the treatment at the indicated concentrations based on live cell imaging. Data points indicate mean from triplicates. Experiments were done at n=2 independent times. (B) Cell cycle analysis in MCF7 and MiaPaCa-2 cells following the treatment with INX-315 (C) Effect of INX-315 on the proliferation of MB157-RB-del cells. Error bars indicate mean and SD from triplicates. Experiment was done at n=3 independent times. (D) Immunoprecipitation of CDK1 in MCF7 and MiaPaCa-2 cells following the treatment with IX-315. The co-immunoprecipitated proteins were determined by western blotting (E) Biolayer interferometry assay of immobilized CDK2 binding to 100 nM Cyclin A in the absence (blue) or presence (red) of 100 nM INX-315. Experiment was done at n=4 biological replicates (F) NanoBRET assay quantifying INX-315's intracellular binding with cyclin A/CDK1 and Cyclin B1/CDK1 complexes. Source data are provided in source data table.

Fig. S6

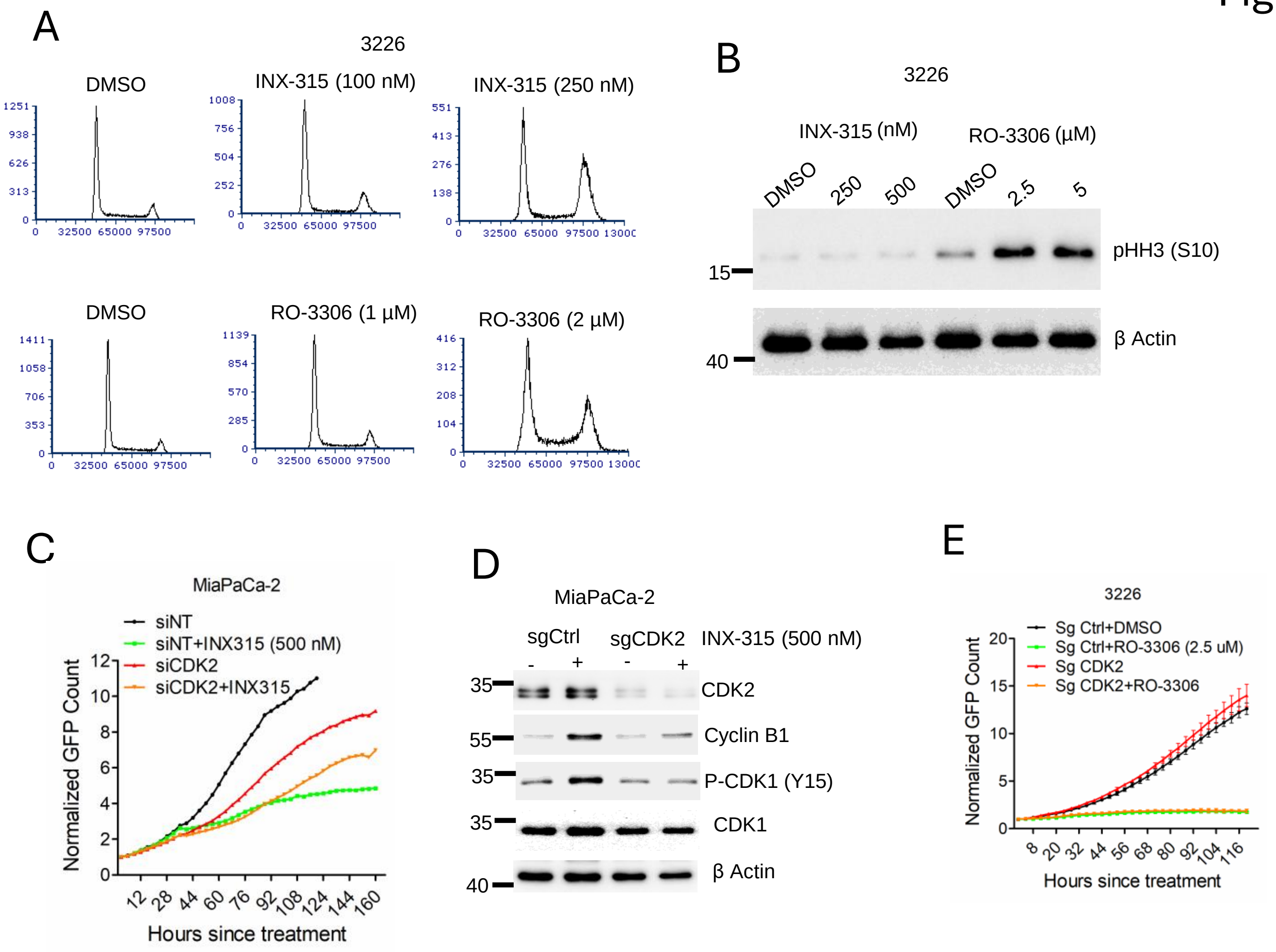

(A) Cell Cycle analysis in 3226 cells following the treatment with INX-315 and RO-3306. (B) Western blotting to determine the effect of INX-315 and RO-3306 on phosphorylation of Histone H3 (S10) in 3226 cells. (C) Proliferation of MiaPaCa-2 cells following the depletion of CDK2 followed by the treatment with INX-315 for the indicated hours. Mean was determined from n=4 technical replicates. Experiment was done at n=2 independent times. (D) Biochemical analysis to determine the differential effect of INX-315 between MiaPaCa-2-sgCtrl and MiaPaCa-sgCDK2 cell lines. (E) Live cell imaging to determine the effect of RO-3306 in 3226 sg-Ctrl and 3226 sg-CDK2 cells. Error bars were calculated based on mean and SD from triplicates. Experiments were done at n=3 independent times. Source data are provided in source data table.

Fig. S7

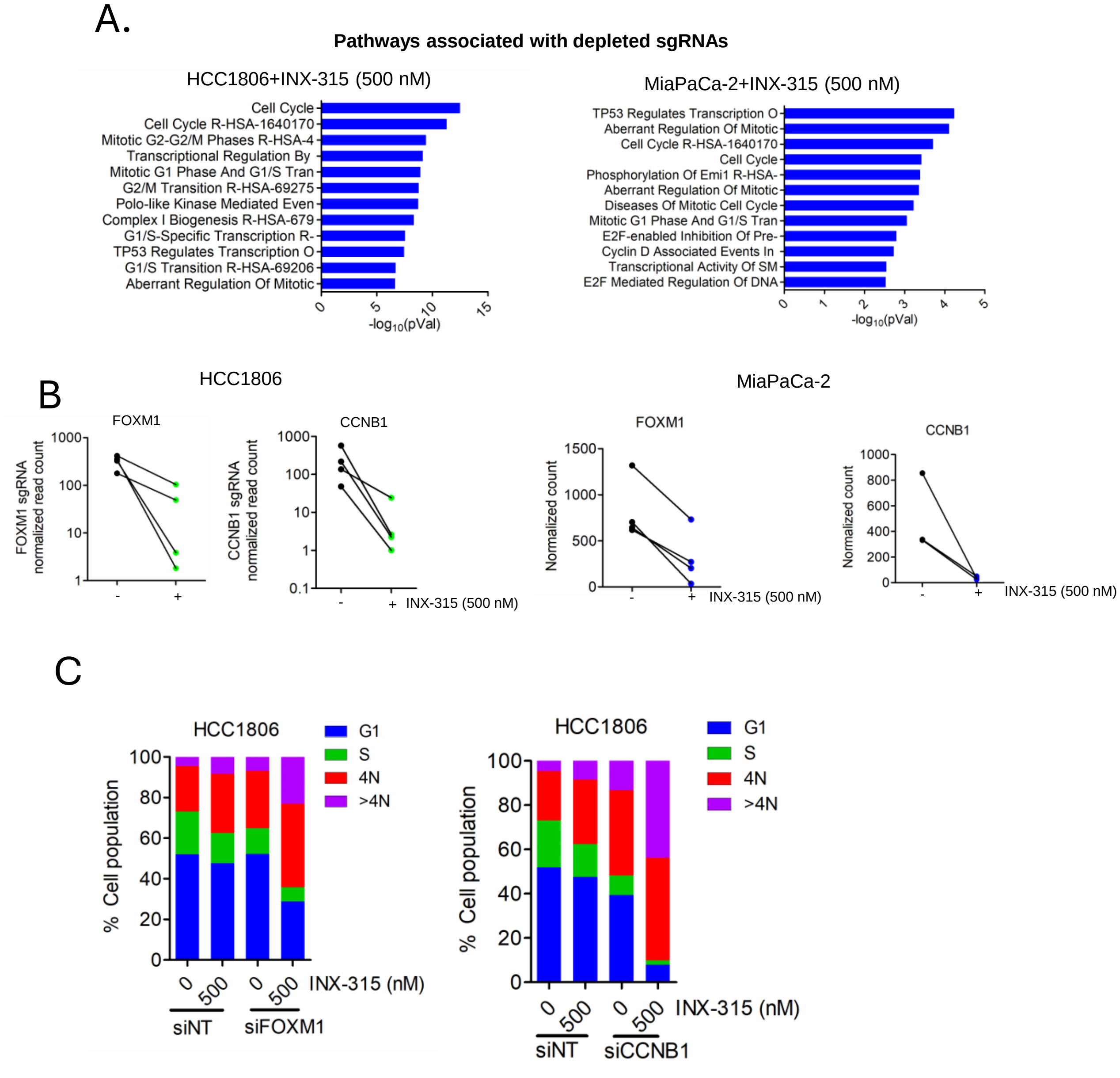

(A) ENRICHR analysis using the genes whose guide sequences were depleted following the INX-315 selection in HCC1806 and MiaPaCa-2 cells. (B) Normalized counts of the individual guides targeting *FOXM1* and *CCNB1* in the absence and presence of INX-315 from MiaPaCa-2 and HCC1806 cells. (C) Population of cells on the indicated phases of cell cycle following FOXM1 and CCNB1 deletion in HCC1806 cells. The data points represent mean from n=2 biological replicates. Source data are provided in source data table.

Fig. S8

1222

Drugs	Target
Barasertib (AZD1152-HQPA)	Aurora Kinase
Alisertib (MLN8237)	Aurora Kinase
Wortmannin	Autophagy,ATM/ATR,PI3K
Palbociclib (PD-0332991) HCl	CDK
BMS-265246	CDK
SNS-032 (BMS-387032)	CDK
MK-8245	Dehydrogenase
Dacomitinib (PF299804, PF299)	EGFR
Afatinib (BIBW2992)	EGFR,HER2
Trichostatin A (TSA)	HDAC
Mubritinib (TAK 165)	HER2
OSI-027	mTOR
WYE-125132 (WYE-132)	mTOR
Rapamycin (Sirolimus)	mTOR,Autophagy
Torkinib (PP242)	mTOR,Autophagy
GSK1059615	PI3K,mTOR
KRN 633	VEGFR,PDGFR

HCC1806

Hesperadin	Aurora Kinase
PHA-793887	CDK
Palbociclib (PD-0332991) HCl	CDK
Milciclib (PHA-848125)	CDK
BMS-265246	CDK
MK-8245	Dehydrogenase
Fluorouracil (5-Fluoracil, 5-FU)	DNA/RNA Synthesis
Dacomitinib (PF299804, PF299)	EGFR
Pimasertib (AS-703026)	MEK
Trametinib (GSK1120212)	MEK
AZD8055	mTOR
WYE-125132 (WYE-132)	mTOR
GDC-0879	Raf

3226

Drugs	Target
PHA-680632	Aurora Kinase
Aurora A Inhibitor I	Aurora Kinase
GSK1070916	Aurora Kinase
MK-5108 (VX-689)	Aurora Kinase
CYC116	Aurora Kinase,VEGFR
ABT-737	Bcl-2,Autophagy
PHA-793887	CDK
Flavopiridol (Alvocidib) HCl	CDK
Palbociclib (PD-0332991) HCl	CDK
WZ3146	EGFR
PD153035 HCl	EGFR
AST-1306	EGFR
AEE788 (NVP-AEE788)	EGFR,HER2,VEGFR
TAE226 (NVP-TAE226)	FAK
PF-03814735	FAK,Aurora Kinase
Entinostat (MS-275)	HDAC
EPZ5676	Histone Methyltransferase
Gandotinib (LY2784544)	JAK
Trametinib (GSK1120212)	MEK
PD0325901	MEK
PD318088	MEK
WYE-354	mTOR
AZD2014	mTOR
Apitolisib (GDC-0980, RG7422)	mTOR,PI3K
PIK-93	PI3K
SB525334	TGF-beta/Smad

List of drug based on drug screening analysis from 1222, 3226 and HCC1806 cells that displayed a cooperative effect with INX-315. 1222 and 3226 cells were treated with INX-315 at 100 nM and HCC1806 cells were treated at 500 nM.

Fig. S9

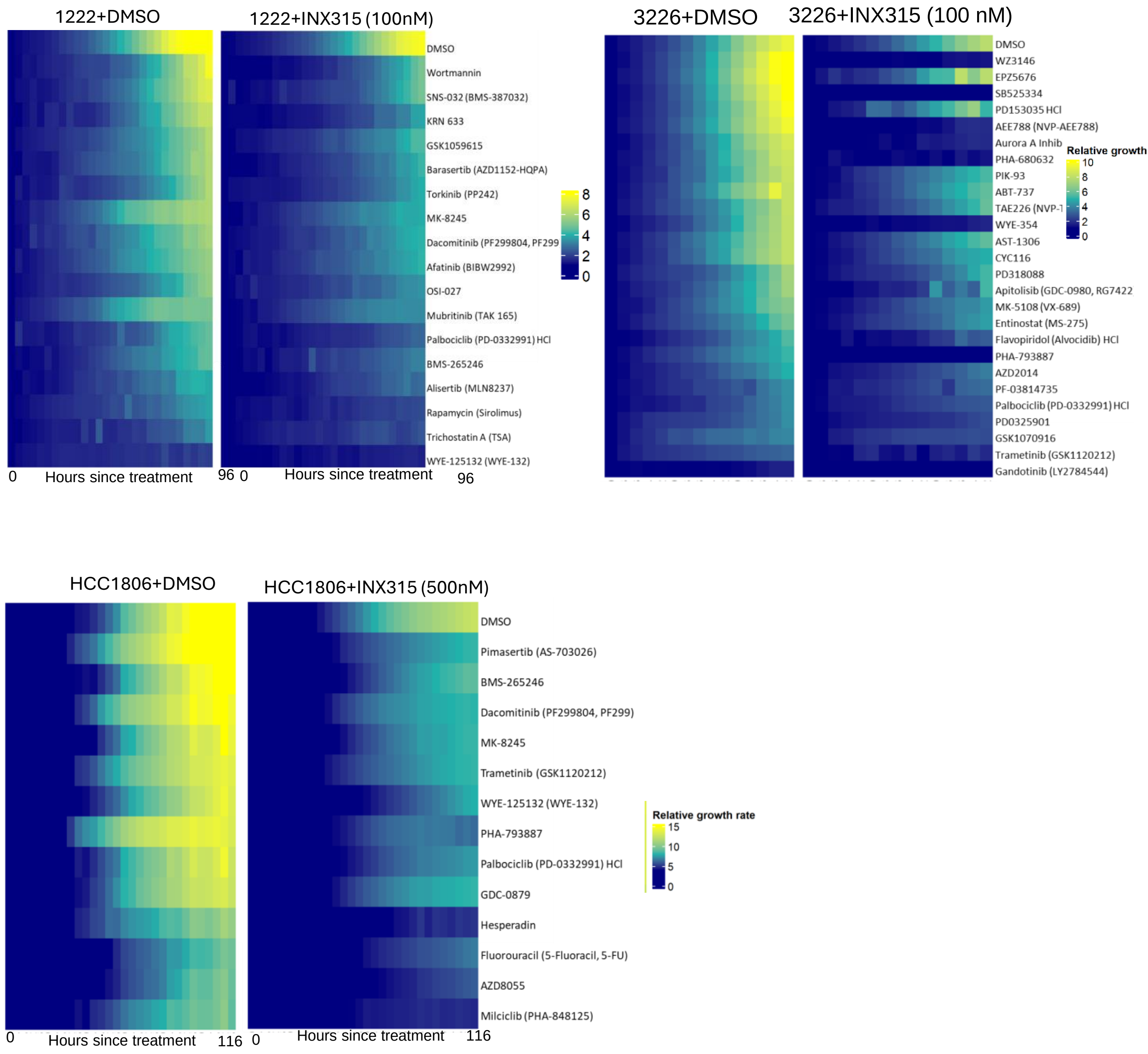

Heatmaps depicting the proliferation of 1222, 3226 and HCC1806 cells in the presence of individual drugs from the library in combination with DMSO and INX-315 over the indicated period of time

Fig. S10

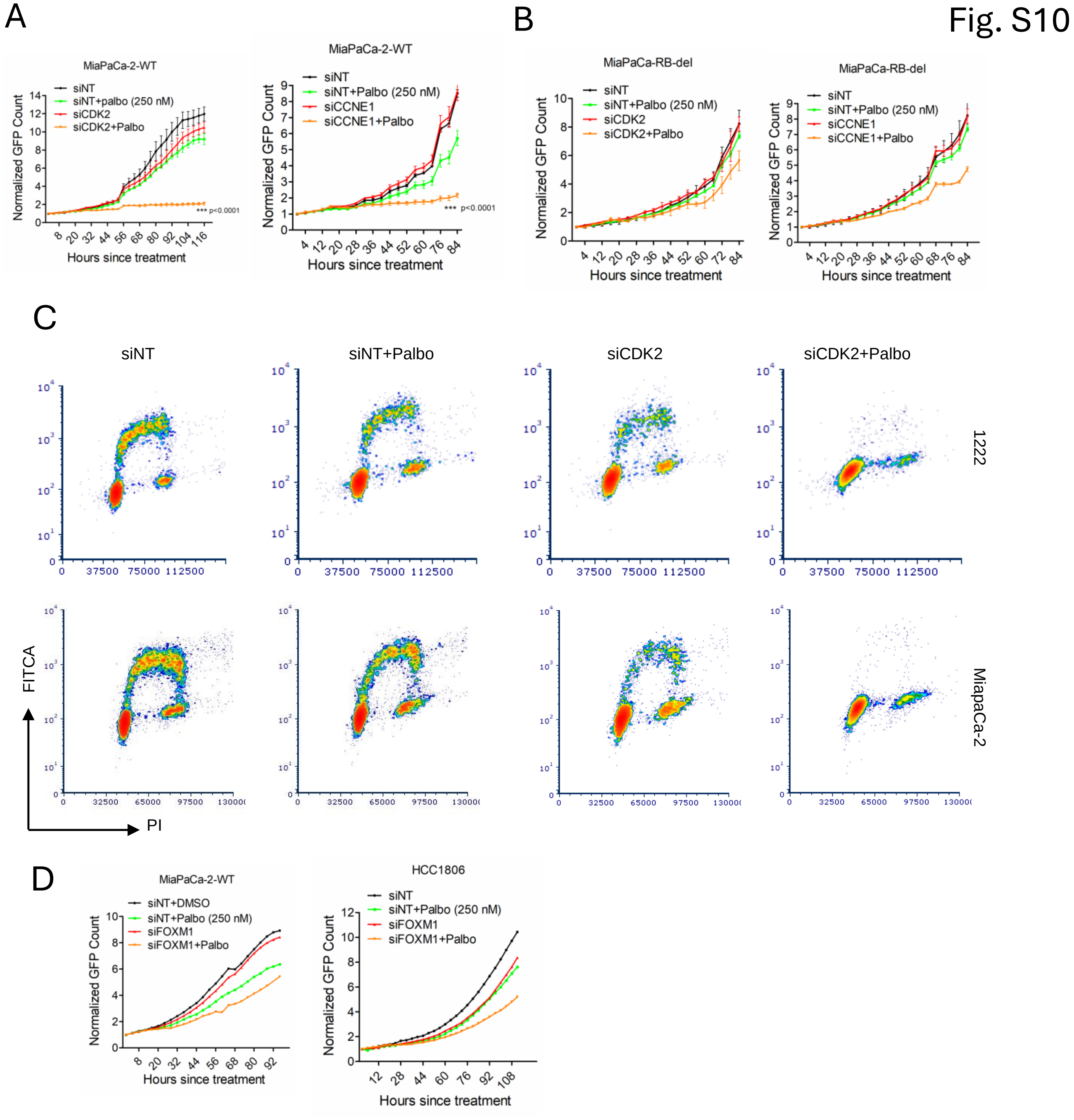

(A) Proliferation of MiaPaCa-2-WT and RB-del cells following the deletion of CDK2 and *CCNE1* in combination with Palbociclib (250 nM). Error bars indicate mean and SD from triplicates. Experiment was done at n=3 independent times. *** represent p value <0.0001 as determined by 2-way ANOVA, comparing siNT+Palbociclib and siCDK2/CCNE1+Palbociclib (B) Effect of *CDK2* and *CCNE1* knockdowns on the growth of MiaPaCa-2-RB-del cells following the treatment with palbociclib. Error bars indicate mean and SD from triplicates. Experiment was done at n=3 independent times. (C) Bi-variate flow cytometry analysis to determine the BrdU incorporation in 1222 and MiaPaCa-2 cells following CDK2 deletion in the absence and presence of palbociclib (250 nM). (D) Effect of FOXM1 deletion in MiaPaCa-2 and HCC1806 cells in the absence and presence of Pallbo. Data points indicate mean from n=4 technical replicates. Source data are provided in source data table.

A

Fig. S11

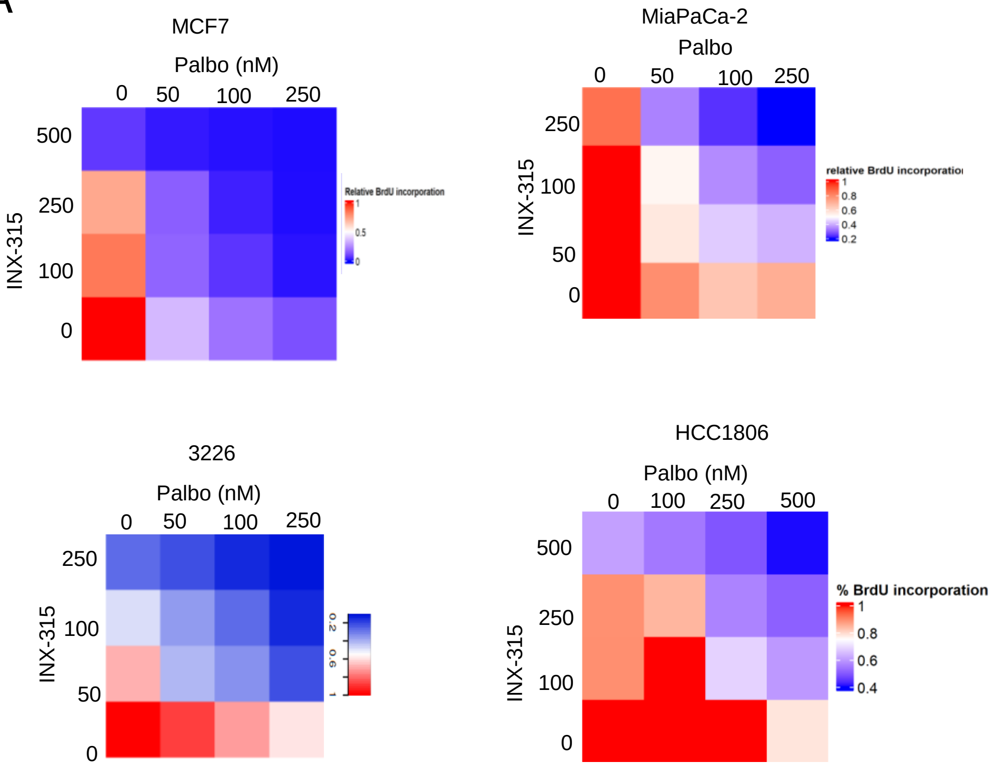

(A) The effect of palbociclib in combination with INX-315 at different concentrations on BrdU incorporation was determined on the indicated cell lines. The heatmaps depict the relative BrdU incorporation following the combination treatment at the indicated doses.

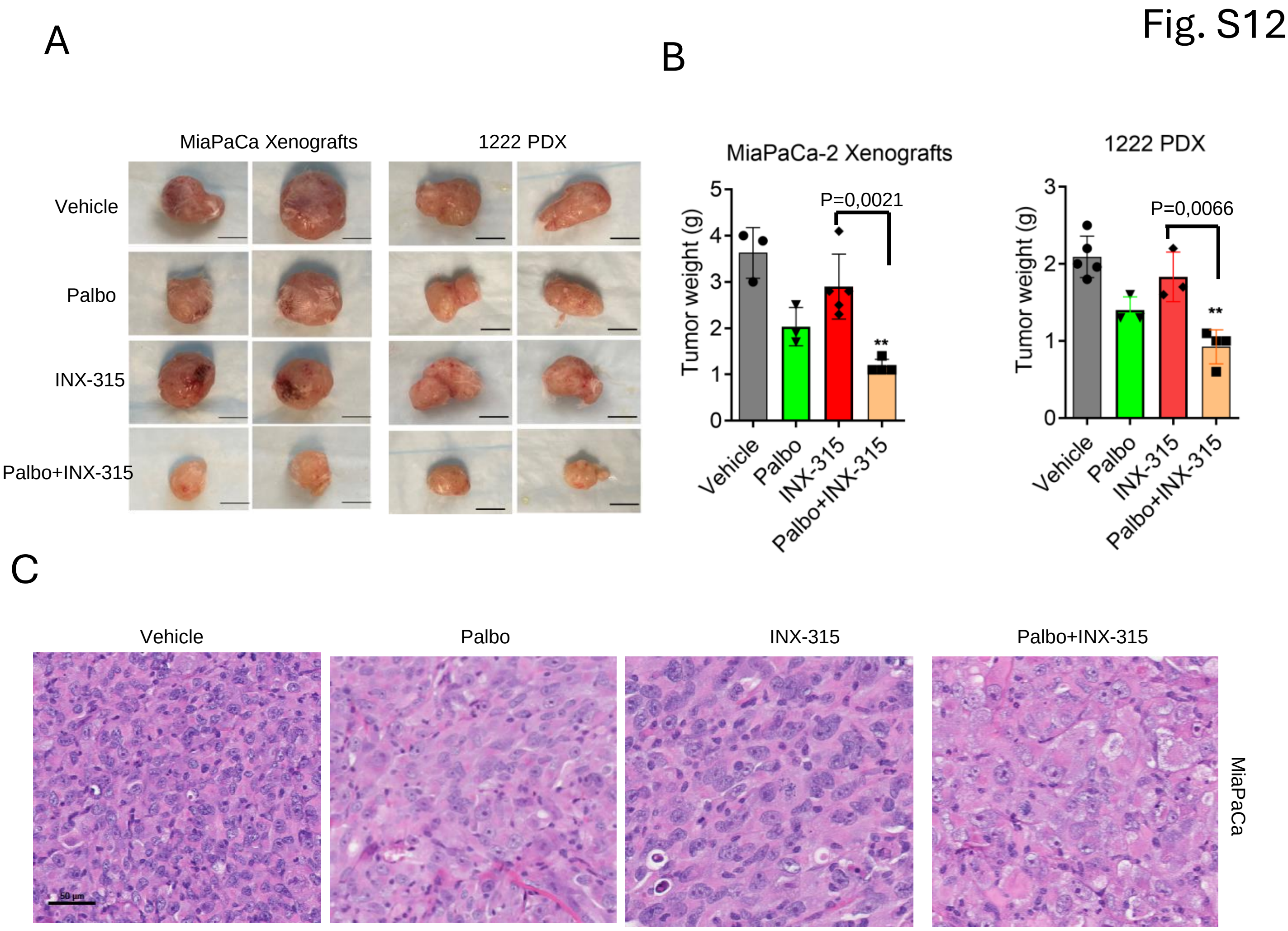

(A) Representative tumor images (n=2 mice) from 1222 PDX and MiaPaCa-2 xenografts that were treated with Vehicle, palbociclib, INX-315 and the Palbociclib+INX-315 groups. (B) Column graphs illustrating the tumor wights from MiaPaCa-2 xenografts and 1222 PDX. The treatment conditions for MiaPaCa-2 xenografts include vehicle (n=3), Palbociclib (n=3), INX-315 (n=5), Palbo+INX-315 (n=4). Error bars represent mean and SEM. ** represent p value = 0.0021 as determined by two-tailed student t test, comparing the INX-315 and Palb+INX-315 treated groups. The treatment conditions for 1222 PDX include, vehicle (n=5), Palbociclib (n=3), INX-315 (n=3), Palbo+INX-315 (n=4). Error bars represent mean and SEM. ** represent p value = 0.0066 as determined by two-tailed student t test, comparing the INX-315 and Palb+INX-315 treated groups. (C) Representative H&E images from MiaPaCa-2 xenografts that were treated with vehicle, palbociclib, INX-315 and palbociclib+INX-315 groups. Scale bar represent 50 microns. Source data are provided in source data table.

Uncropped Supplementary Images

Fig. S1G

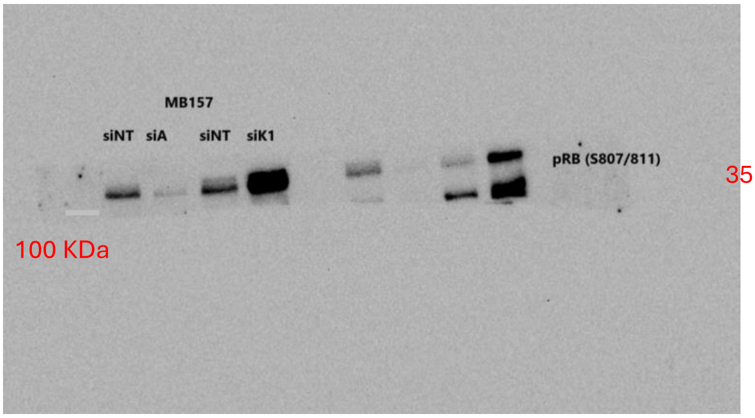
MB157
siNT siA siNT siK1
pRB (S807/811)
100 KDa

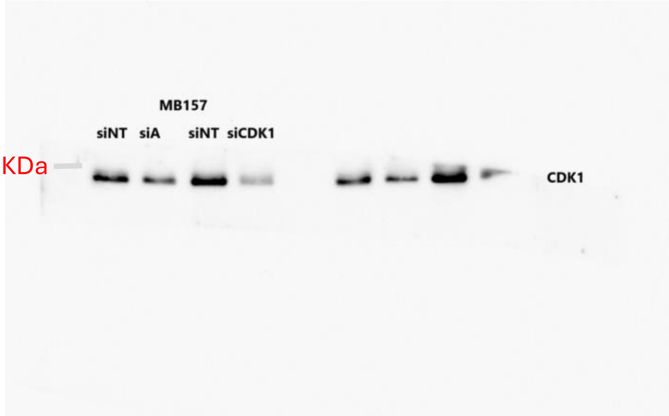
MB157
siNT siA siNT siCDK1
35 KDa
CDK1

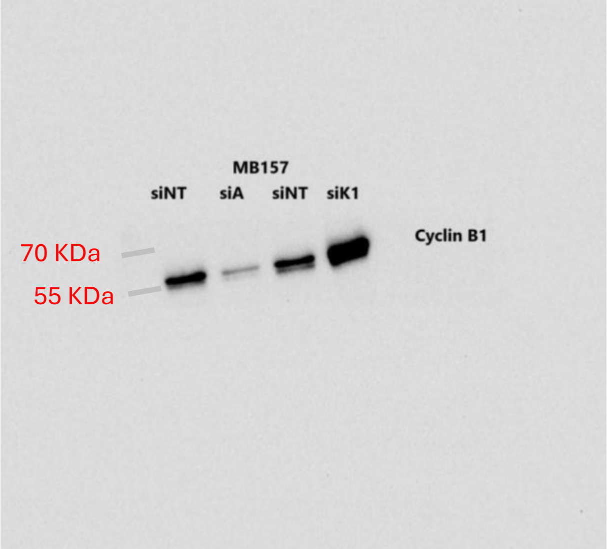
MB157
siNT siA siNT siK1
70 KDa
55 KDa
Cyclin B1

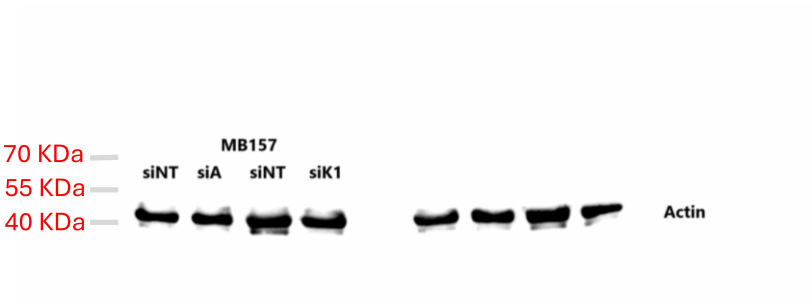
70 KDa
55 KDa
40 KDa
MB157
siNT siA siNT siK1
Actin

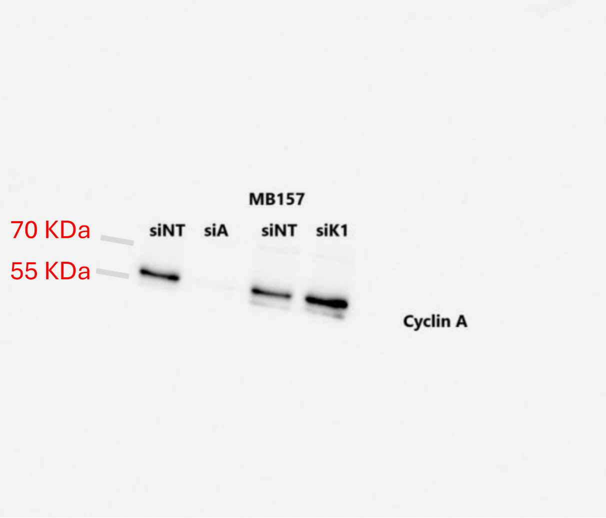
MB157
70 KDa
55 KDa
siNT siA siNT siK1
Cyclin A

Fig. S3D

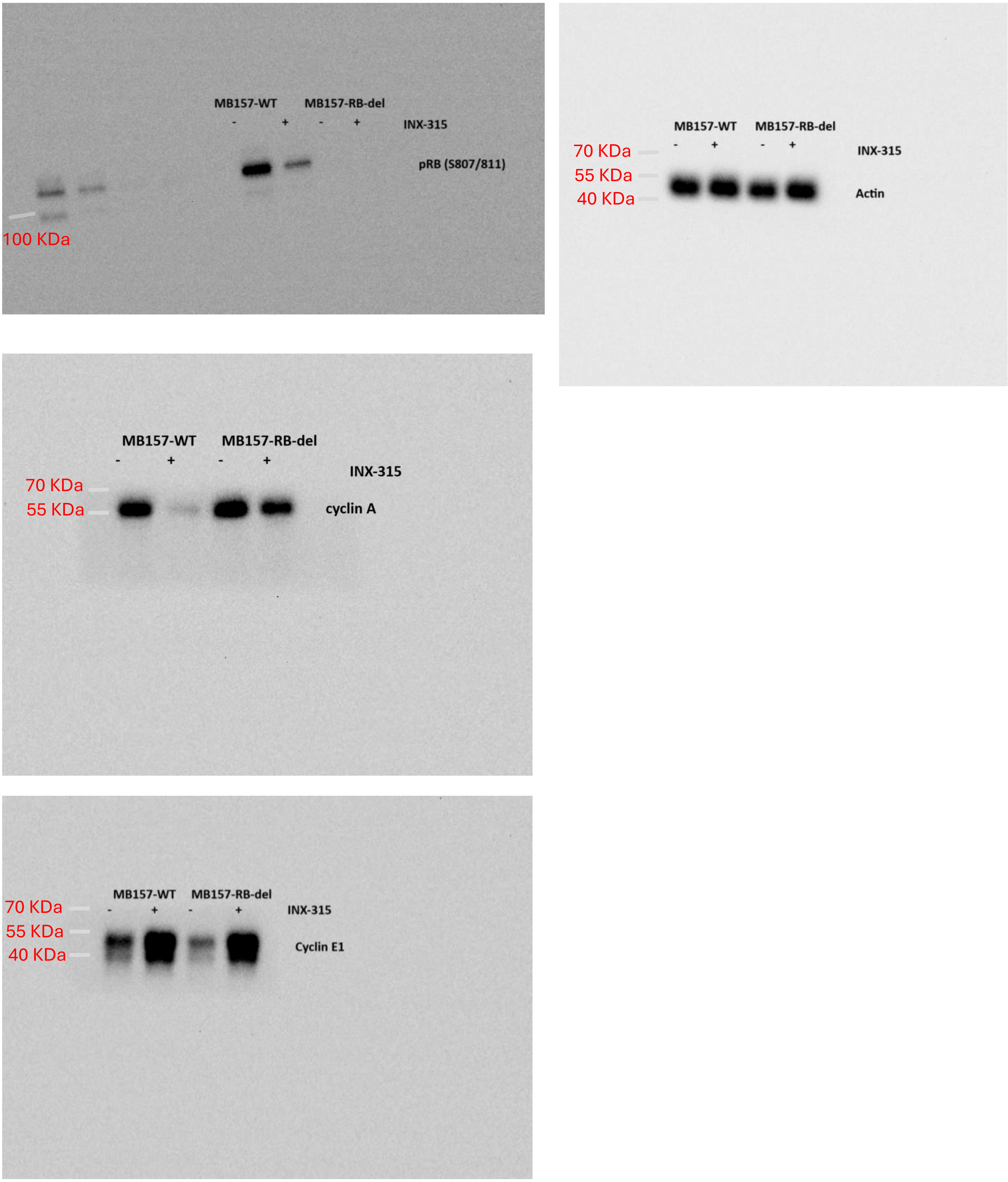
MB157-WT
MB157-RB-del
- + - +
INX-315
pRB (S807/811)
100 KDa
MB157-WT
MB157-RB-del
- + - +
INX-315
70 KDa
55 KDa
40 KDa
Actin
MB157-WT
MB157-RB-del
- + - +
INX-315
70 KDa
55 KDa
cyclin A
MB157-WT
MB157-RB-del
- + - +
INX-315
70 KDa
55 KDa
40 KDa
Cyclin E1

Fig. S3F

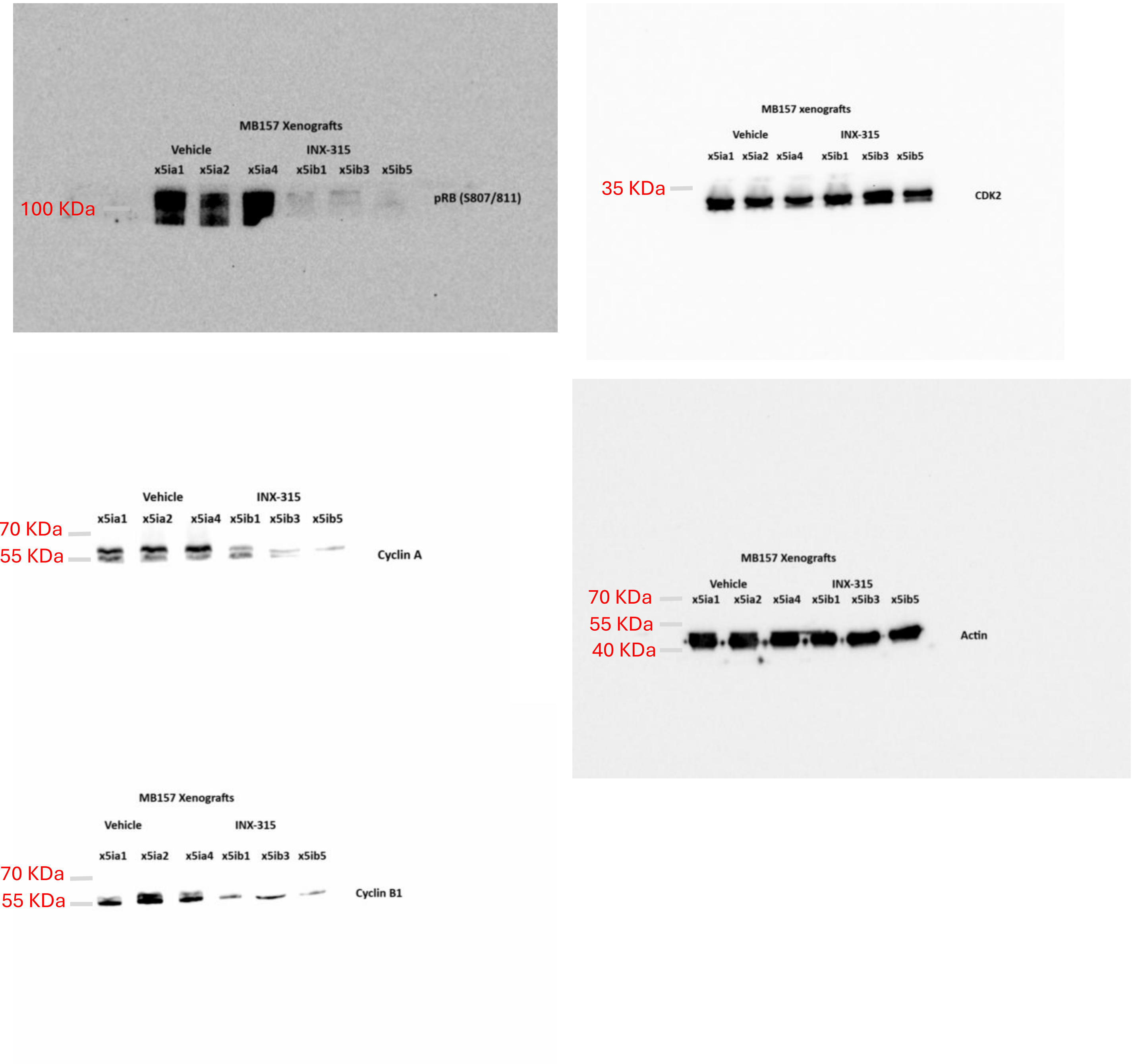
MB157 Xenografts
Vehicle
INX-315
x5ia1 x5ia2 x5ia4 x5ib1 x5ib3 x5ib5
100 KDa
pRB (S807/811)
MB157 xenografts
Vehicle
INX-315
x5ia1 x5ia2 x5ia4 x5ib1 x5ib3 x5ib5
35 KDa
CDK2
Vehicle
INX-315
x5ia1 x5ia2 x5ia4 x5ib1 x5ib3 x5ib5
70 KDa
55 KDa
Cyclin A
MB157 Xenografts
Vehicle
INX-315
x5ia1 x5ia2 x5ia4 x5ib1 x5ib3 x5ib5
70 KDa
55 KDa
40 KDa
Actin
MB157 Xenografts
Vehicle
INX-315
x5ia1 x5ia2 x5ia4 x5ib1 x5ib3 x5ib5
70 KDa
55 KDa
Cyclin B1

Fig. S4C

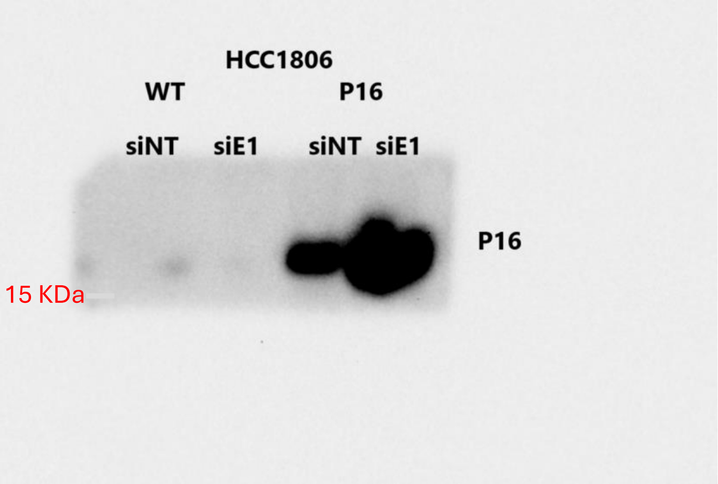
HCC1806
WT
P16
siNT
siE1
siNT
siE1
P16
15 KDa

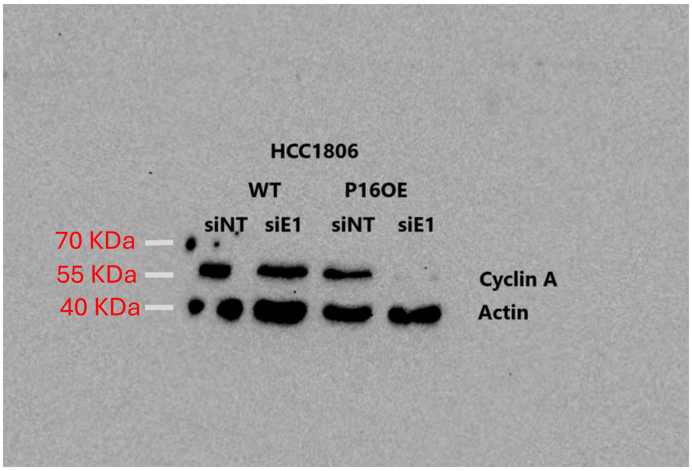
HCC1806
WT
P16OE
siNT
siE1
siNT
siE1
70 KDa
55 KDa
40 KDa
Cyclin A
Actin

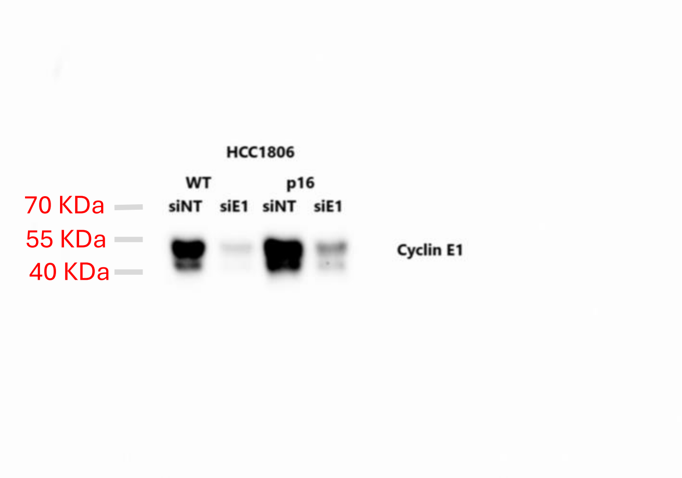
HCC1806
WT
p16
siNT
siE1
siNT
siE1
70 KDa
55 KDa
40 KDa
Cyclin E1

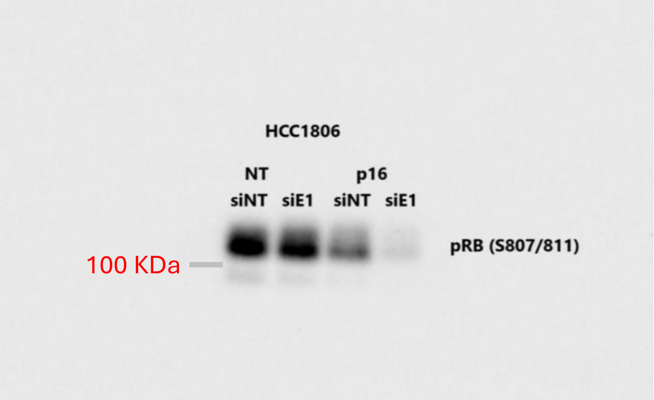
HCC1806
NT
p16
siNT
siE1
siNT
siE1
100 KDa
pRB (S807/811)

Fig. S4C

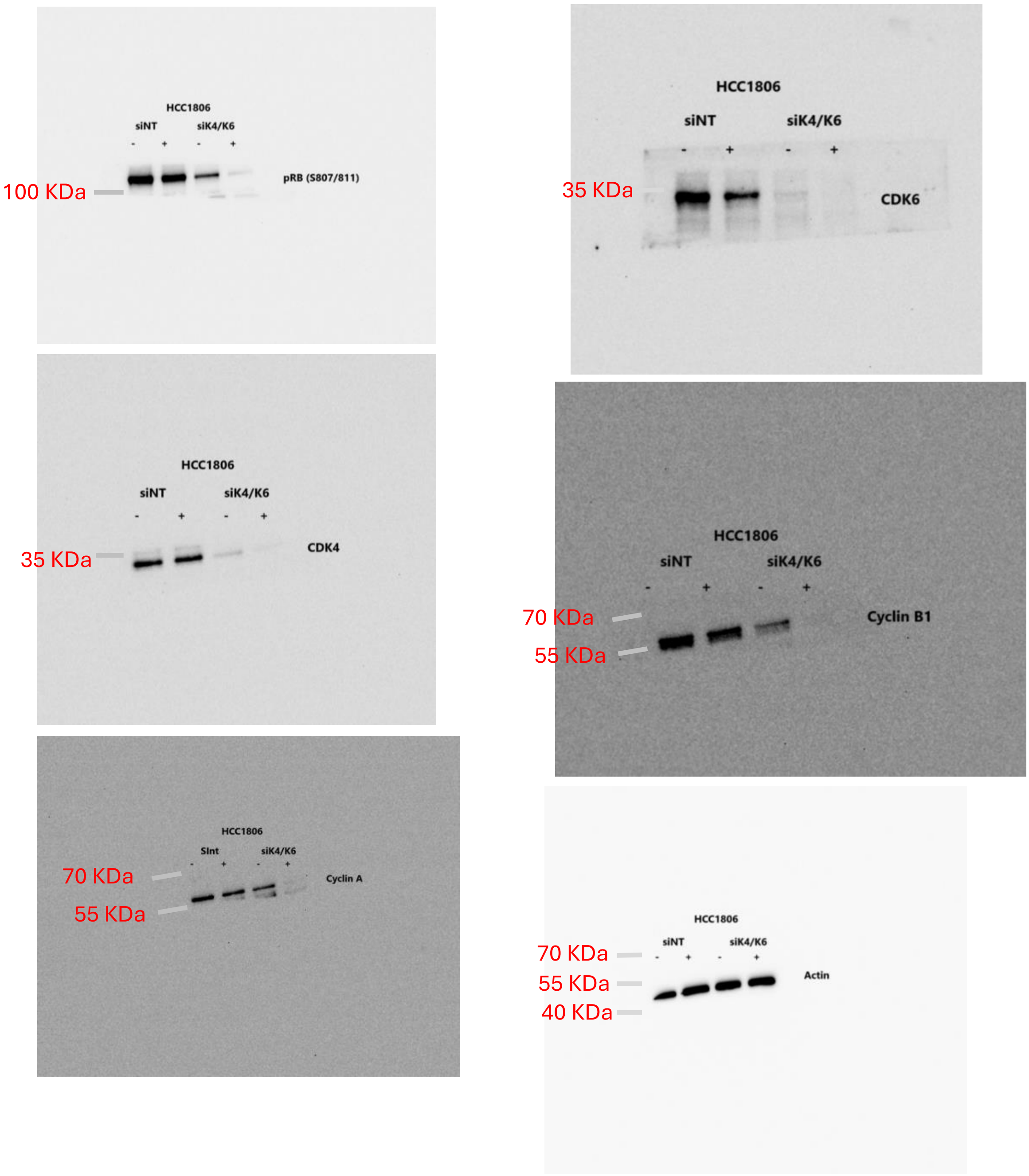
HCC1806
siNT
siK4/K6
100 KDa
pRB (S807/811)
HCC1806
siNT
siK4/K6
35 KDa
CDK6
HCC1806
siNT
siK4/K6
35 KDa
CDK4
HCC1806
siNT
siK4/K6
70 KDa
55 KDa
Cyclin B1
HCC1806
SInt
siK4/K6
70 KDa
55 KDa
Cyclin A
HCC1806
siNT
siK4/K6
70 KDa
55 KDa
40 KDa
Actin

Fig. S4E

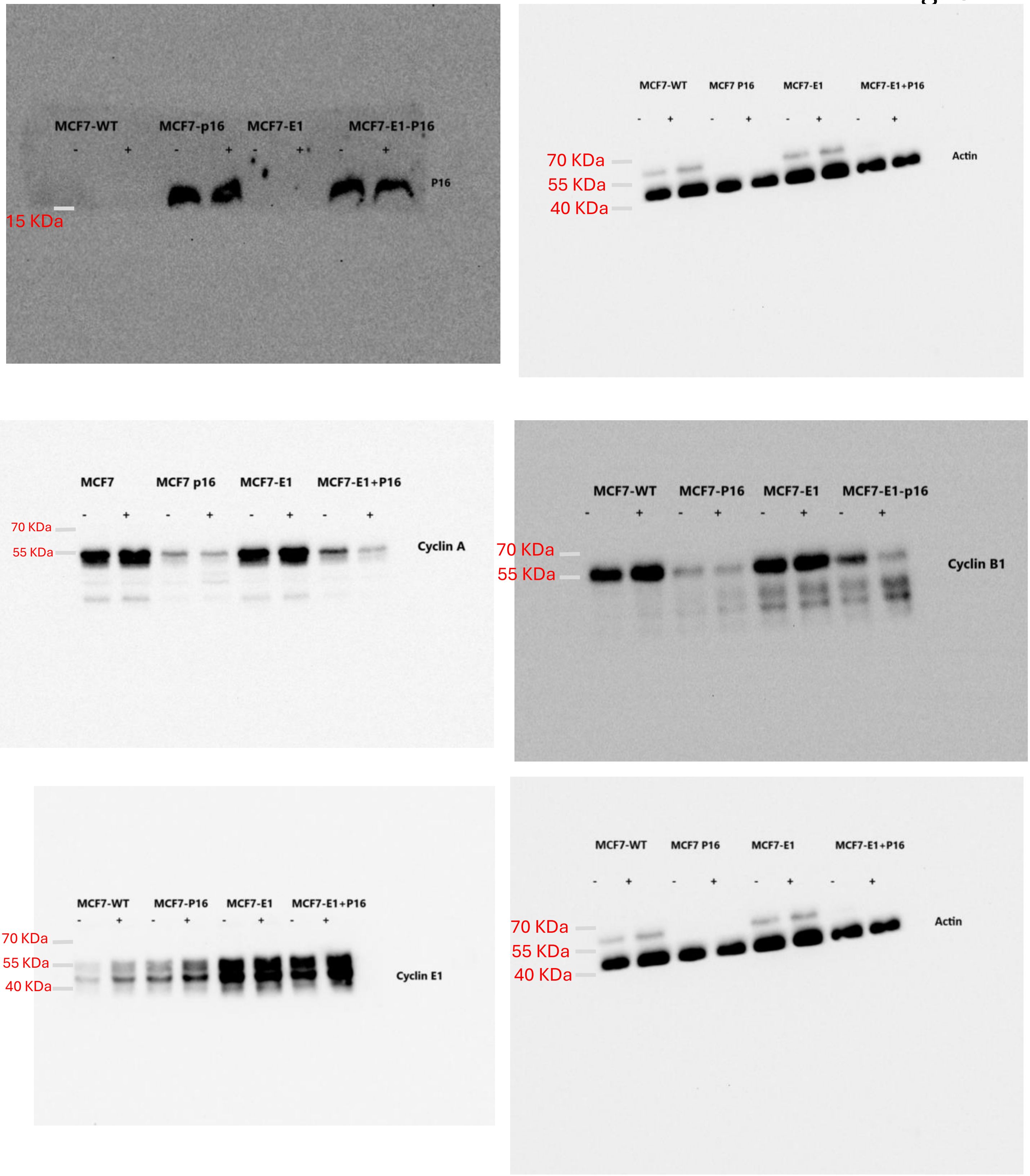

MCF7-WT
MCF7-p16
MCF7-E1
MCF7-E1-P16
- + - + - + - +
P16
15 KDa
MCF7-WT
MCF7 P16
MCF7-E1
MCF7-E1+P16
- + - + - + - +
70 KDa
55 KDa
40 KDa
Actin
MCF7
MCF7 p16
MCF7-E1
MCF7-E1+P16
- + - + - + - +
70 KDa
55 KDa
Cyclin A
MCF7-WT
MCF7-P16
MCF7-E1
MCF7-E1-p16
- + - + - + - +
70 KDa
55 KDa
Cyclin B1
MCF7-WT
MCF7-P16
MCF7-E1
MCF7-E1+P16
- + - + - + - +
70 KDa
55 KDa
40 KDa
Cyclin E1
MCF7-WT
MCF7 P16
MCF7-E1
MCF7-E1+P16
- + - + - + - +
70 KDa
55 KDa
40 KDa
Actin

Fig. S5D

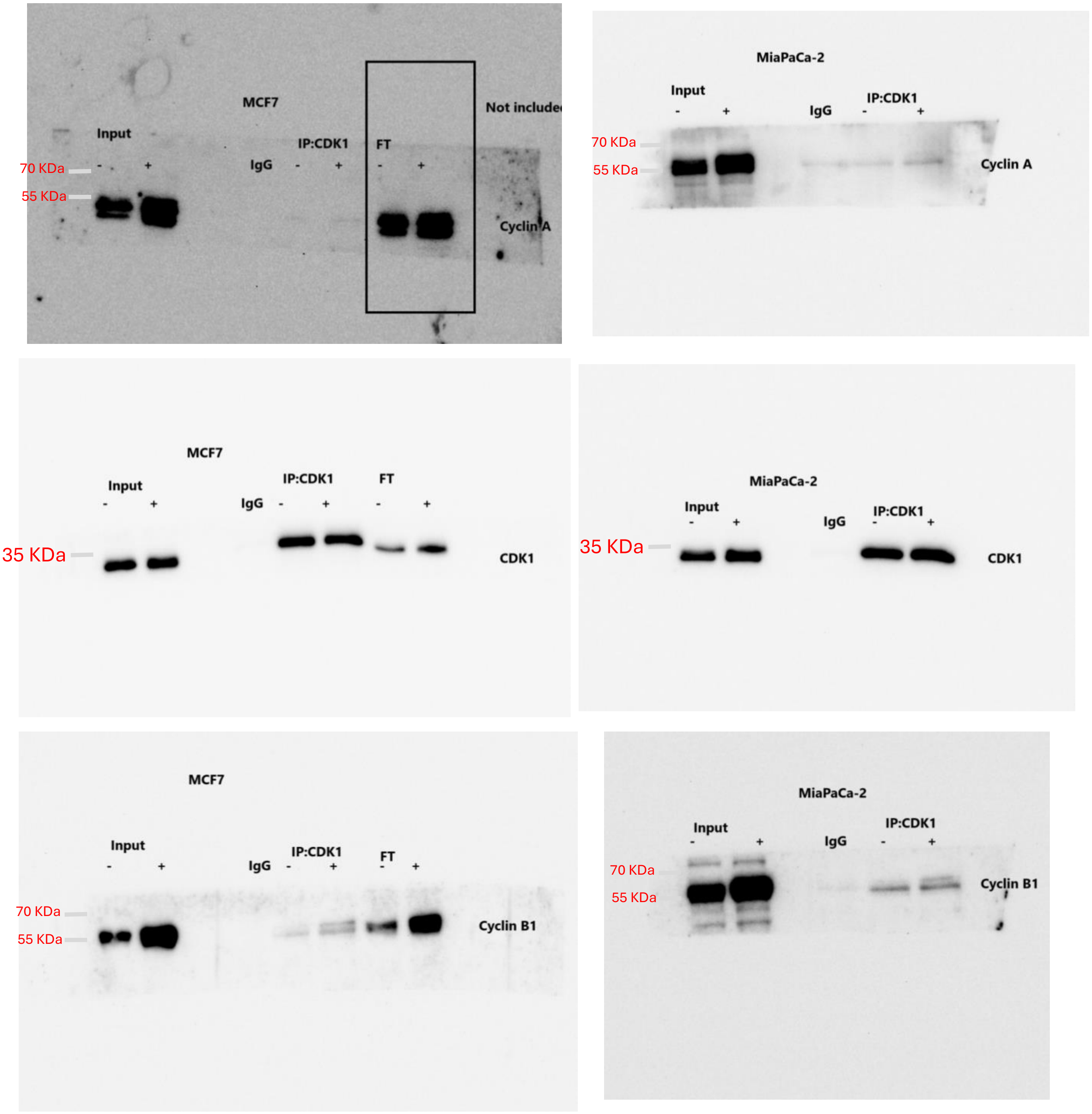

MCF7
Input
IP:CDK1
FT
Not include
IgG
70 KDa
55 KDa
Cyclin A
MiaPaCa-2
Input
IP:CDK1
IgG
70 KDa
55 KDa
Cyclin A
MCF7
Input
IP:CDK1
FT
IgG
35 KDa
CDK1
MiaPaCa-2
Input
IP:CDK1
IgG
35 KDa
CDK1
MCF7
Input
IP:CDK1
FT
IgG
70 KDa
55 KDa
Cyclin B1
MiaPaCa-2
Input
IP:CDK1
IgG
70 KDa
55 KDa
Cyclin B1

Fig. S6B

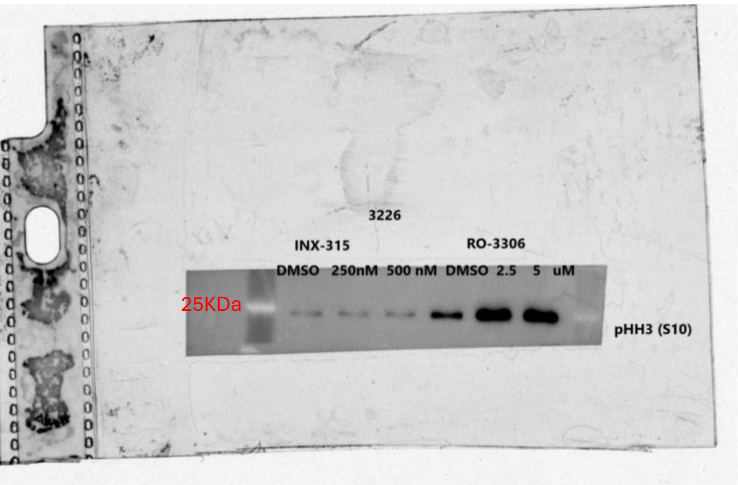

3226
INX-315
RO-3306
DMSO 250nM 500 nM DMSO 2.5 5 uM
25KDa
pHH3 (S10)

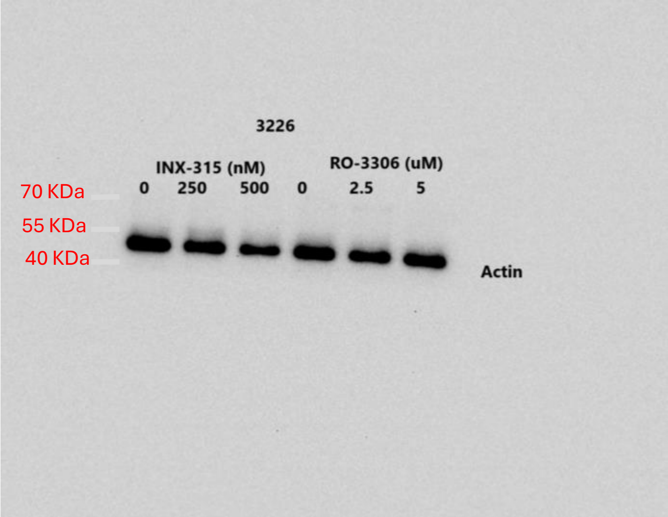

3226
INX-315 (nM)
RO-3306 (uM)
0 250 500 0 2.5 5
70 KDa
55 KDa
40 KDa
Actin

Fig. S6D

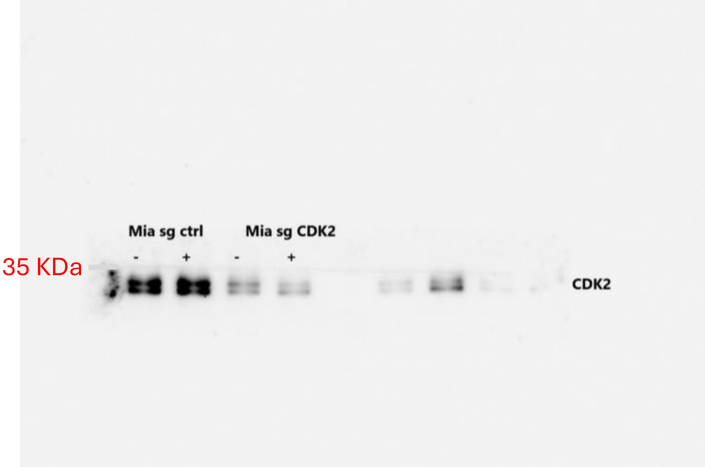

Mia sg ctrl
Mia sg CDK2
- + - +
35 KDa
CDK2

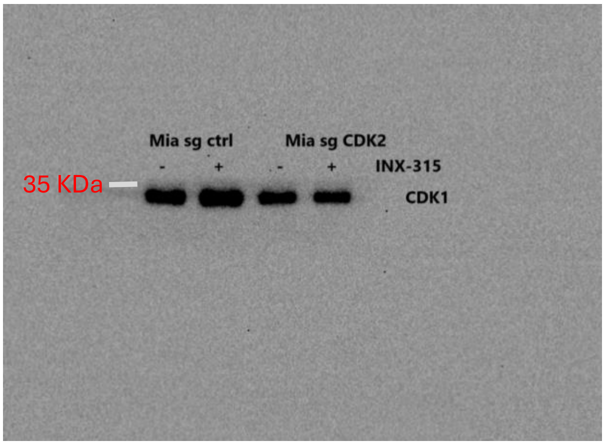

Mia sg ctrl
Mia sg CDK2
- + - +
INX-315
35 KDa
CDK1

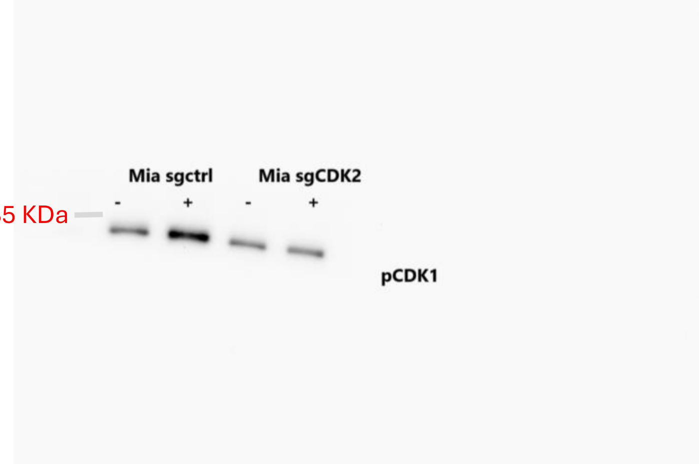

Mia sgctrl
Mia sgCDK2
- + - +
35 KDa
pCDK1

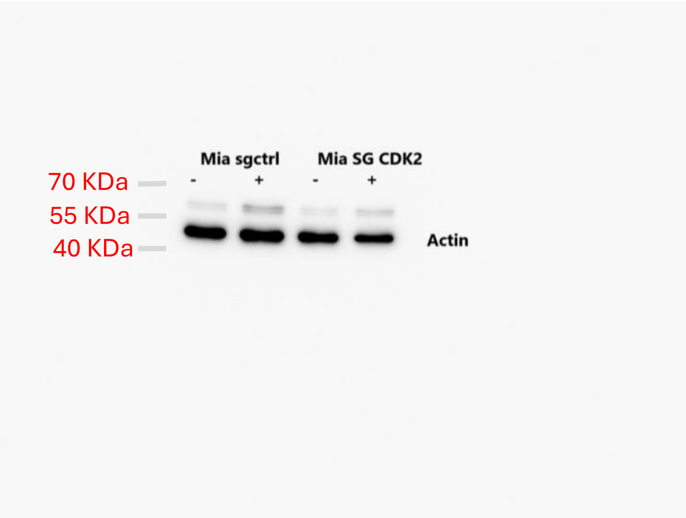

Mia sgctrl
Mia SG CDK2
- + - +
70 KDa
55 KDa
40 KDa
Actin

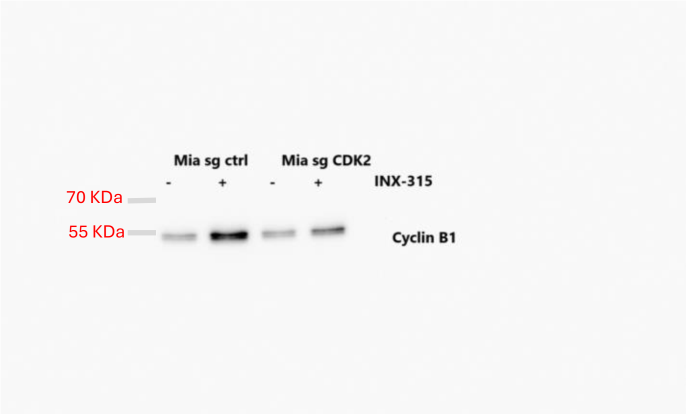

Mia sg ctrl
Mia sg CDK2
- + - +
INX-315
70 KDa
55 KDa
Cyclin B1

Author contributions:

Study concepts and design: VK, CF, AT, PR, SR, KM, ESK and AKW

Acquisition of data: VK, CF, JW, YW, AD, HR, AT, JB, JS, PR and SP

Analysis and interpretation of data: VK, CF, JW, YW, AD, HR, SP, AT, PR, ESK and AKW

Study supervision: ESK and AKW.
