# Supplementary material for: Discrete vulnerability to pharmacological CDK2 inhibition is governed by heterogeneity of the cancer cell cycle
Source: Nat Commun. 2025 Feb 9;16:1476. doi: 10.1038/s41467-025-56674-4 (PMC11808123; doi:10.1038/s41467-025-56674-4)
Supplement: Supplementary file 2 — Reporting Summary [file 41467_2025_56674_MOESM2_ESM.pdf]

nature portfolio

Corresponding author(s): Erik Knudsen

Last updated by author(s): 12/24/24

Reporting Summary

Nature Portfolio wishes to improve the reproducibility of the work that we publish. This form provides structure for consistency and transparency in reporting. For further information on Nature Portfolio policies, see our Editorial Policies and the Editorial Policy Checklist.

Please do not complete any field with "not applicable" or n/a. Refer to the help text for what text to use if an item is not relevant to your study.
For final submission: please carefully check your responses for accuracy; you will not be able to make changes later.

Statistics

For all statistical analyses, confirm that the following items are present in the figure legend, table legend, main text, or Methods section.

n/a	Confirmed	
☐	☑	The exact sample size (n) for each experimental group/condition, given as a discrete number and unit of measurement
☐	☑	A statement on whether measurements were taken from distinct samples or whether the same sample was measured repeatedly
☐	☑	The statistical test(s) used AND whether they are one- or two-sided<br>*Only common tests should be described solely by name; describe more complex techniques in the Methods section.*
☑	☐	A description of all covariates tested
☐	☑	A description of any assumptions or corrections, such as tests of normality and adjustment for multiple comparisons
☐	☑	A full description of the statistical parameters including central tendency (e.g. means) or other basic estimates (e.g. regression coefficient) AND variation (e.g. standard deviation) or associated estimates of uncertainty (e.g. confidence intervals)
☑	☐	For null hypothesis testing, the test statistic (e.g. F, t, r) with confidence intervals, effect sizes, degrees of freedom and P value noted<br>*Give P values as exact values whenever suitable.*
☑	☐	For Bayesian analysis, information on the choice of priors and Markov chain Monte Carlo settings
☑	☐	For hierarchical and complex designs, identification of the appropriate level for tests and full reporting of outcomes
☐	☑	Estimates of effect sizes (e.g. Cohen's d, Pearson's r), indicating how they were calculated

Our web collection on statistics for biologists contains articles on many of the points above.

Software and code

Policy information about availability of computer code

Data collection	IncuCyte S3 and CellCytex were used to get the cell counts in real time. Biorad imager was used to get the western blot images. FORTESSA was used to detemine the population of cells at each phases ofcell cycle
Data analysis	GraphPad Prism was used to generate all data charts. FCS express was used to analyze the flowcytometry data.EdgeR package was used for differential gene expression analysis . Biorad Image Lab software was used to process the western blot images

For manuscripts utilizing custom algorithms or software that are central to the research but not yet described in published literature, software must be made available to editors and reviewers. We strongly encourage code deposition in a community repository (e.g. GitHub). See the Nature Portfolio guidelines for submitting code & software for further information.

Data

Policy information about availability of data

All manuscripts must include a data availability statement. This statement should provide the following information, where applicable:

- Accession codes, unique identifiers, or web links for publicly available datasets
- A description of any restrictions on data availability
- For clinical datasets or third party data, please ensure that the statement adheres to our policy

We have included the data availability statement in the manuscript. The source data are provided with this manuscript. Other data sets that were generated are submitted as supplementary data,

Research involving human participants, their data, or biological material

Policy information about studies with human participants or human data. See also policy information about sex, gender (identity/presentation), and sexual orientation and race, ethnicity and racism.

Reporting on sex and gender	This study includes both men and women
Reporting on race, ethnicity, or other socially relevant groupings	NA
Population characteristics	NA
Recruitment	The participants were prospectively consented to participate in the study
Ethics oversight	IRB at Roswell Park Cancer Center

Note that full information on the approval of the study protocol must also be provided in the manuscript.

Field-specific reporting

Please select the one below that is the best fit for your research. If you are not sure, read the appropriate sections before making your selection.

☑ Life sciences ☐ Behavioural & social sciences ☐ Ecological, evolutionary & environmental sciences

For a reference copy of the document with all sections, see nature.com/documents/nr-reporting-summary-flat.pdf

Life sciences study design

All studies must disclose on these points even when the disclosure is negative.

Sample size	All the plate based assays were performed in triplicate (n=3 technical replicates) and the experiment was performed at three independent times to validate the statistical significance. The western blotting were performed at two independent times.
Data exclusions	Data exclusions were done in mice experiments. Mice that died during the course of treatment, were excluded from the analysis. O ther than that all data were included.
Replication	All the experiments were replicated to ensure that the outcome is reproducible and consistent.
Randomization	Mice were randomized to treatment groups by having the same average tumor size across multiple groups. For cell culture experiments, all cells were pooled together and then randomly split into different groups to minimize variations.
Blinding	Blinding is not applicable for this study. This study involves preclinical investigation where the investigator is aware of the experimental design and different groups.

Behavioural & social sciences study design

All studies must disclose on these points even when the disclosure is negative.

Study description	
Research sample	
Sampling strategy	
Data collection	
Timing	
Data exclusions	
Non-participation	
Randomization	

Ecological, evolutionary & environmental sciences study design

All studies must disclose on these points even when the disclosure is negative.

Study description

Research sample

Sampling strategy

Data collection

Timing and spatial scale

Data exclusions

Reproducibility

Randomization

Blinding

Did the study involve field work? ☐ Yes ☐ No

Field work, collection and transport

Field conditions

Location

Access & import/export

Disturbance

Reporting for specific materials, systems and methods

We require information from authors about some types of materials, experimental systems and methods used in many studies. Here, indicate whether each material, system or method listed is relevant to your study. If you are not sure if a list item applies to your research, read the appropriate section before selecting a response.

Materials & experimental systems

n/a	Involved in the study
☐	☑ Antibodies
☐	☑ Eukaryotic cell lines
☑	☐ Palaeontology and archaeology
☐	☑ Animals and other organisms
☐	☑ Clinical data
☑	☐ Dual use research of concern
☑	☐ Plants

Methods

n/a	Involved in the study
☐	☐ ChIP-seq
☐	☑ Flow cytometry
☐	☐ MRI-based neuroimaging

Antibodies

Antibodies used

pRB (S807/811) (D20B12) (Cat # 8516S), Cyclin B1 (D5C10) (Cat # 12231S), CDK4 (D9G3E) (Cat # 127 90S), CDK2 (78B2) (Cat # 2546S), Cyclin E1 (HE12) (Cat # 4129S), pCDK1 (Y15) (9111L), CDK1 (Cat # 7 7055), P16INK4A (D7C1M) (Cat# 80772) , FOXM1 (D12D5) (Cat # 5436S), Cyclin A (Cat # AF5999) and β Actin (Cat #MAB8929)

Validation

To validate that the antibody recognizes the specific protein, we used gene specific RNAi to delete the target protein. Western blot analysis indicates a reduction in the signal corresponding to the expected molecular weight of the protein, confirming that the antibody recognizes the specific protein.

Eukaryotic cell lines

Policy information about cell lines and Sex and Gender in Research

Cell line source(s)	MCF7 (ATCC, HTB-22), CAMA-1 (ATCC, HTB-21), MIA PaCa-2 (ATCC, CRM-CRL-1420), MB157 (ATCC, HTB-24), HCC1806 (ATCC, CRL-2335). KURAMOCHI cells were provided by Dr. Karen McLean from Roswell Park Cancer Center, 1222 and 3226 are patients-derived PDAC cell lines generated in our lab We could not get the gender information
Authentication	All cell lines were authenticated using STR profiling
Mycoplasma contamination	All cell lines were tested to be Mycoplasma free based on PCR analysis and DAPI staining
Commonly misidentified lines (See ICLAC register)	There are no misidentified cell lines in this study.

Palaeontology and Archaeology

Specimen provenance	NA
Specimen deposition	NA
Dating methods	NA

☐ Tick this box to confirm that the raw and calibrated dates are available in the paper or in Supplementary Information.

Ethics oversight	NA

Note that full information on the approval of the study protocol must also be provided in the manuscript.

Animals and other research organisms

Policy information about studies involving animals; ARRIVE guidelines recommended for reporting animal research, and Sex and Gender in Research

Laboratory animals	NSG mice
Wild animals	NA
Reporting on sex	Both female and male mice were used. The detailed information on the gender of the mice were included in the methods section.
Field-collected samples	NA
Ethics oversight	IACUC at Roswell Park Cancer Center

Note that full information on the approval of the study protocol must also be provided in the manuscript.

Clinical data

Policy information about clinical studies

All manuscripts should comply with the ICMJE guidelines for publication of clinical research and a completed CONSORT checklist must be included with all submissions.

Clinical trial registration	NCT04526587
Study protocol	ClinicalTrials.gov
Data collection	All patient data was collected from a review of patient charts at Roswell Park Comprehensive Cancer Center between July 2020 and December 2022
Outcomes	PFS and OS were based on data from electronic medical record

Dual use research of concern

Policy information about dual use research of concern

Hazards

Could the accidental, deliberate or reckless misuse of agents or technologies generated in the work, or the application of information presented in the manuscript, pose a threat to:

No	Yes	
☑	☐	Public health
☑	☐	National security
☑	☐	Crops and/or livestock
☑	☐	Ecosystems
☑	☐	Any other significant area

Experiments of concern

Does the work involve any of these experiments of concern:

No	Yes	
☑	☐	Demonstrate how to render a vaccine ineffective
☑	☐	Confer resistance to therapeutically useful antibiotics or antiviral agents
☑	☐	Enhance the virulence of a pathogen or render a nonpathogen virulent
☑	☐	Increase transmissibility of a pathogen
☑	☐	Alter the host range of a pathogen
☑	☐	Enable evasion of diagnostic/detection modalities
☑	☐	Enable the weaponization of a biological agent or toxin
☑	☐	Any other potentially harmful combination of experiments and agents

Plants

Seed stocks	NA
Novel plant genotypes	NA
Authentication	NA

ChIP-seq

Data deposition

☐ Confirm that both raw and final processed data have been deposited in a public database such as GEO.

☐ Confirm that you have deposited or provided access to graph files (e.g. BED files) for the called peaks.

Data access links *May remain private before publication.*	
Files in database submission	
Genome browser session (e.g. UCSC)	

Methodology

Replicates	
Sequencing depth	
Antibodies	
Peak calling parameters	
Data quality	

Software

Flow Cytometry

Plots

Confirm that:

- [x] The axis labels state the marker and fluorochrome used (e.g. CD4-FITC).
- [x] The axis scales are clearly visible. Include numbers along axes only for bottom left plot of group (a 'group' is an analysis of identical markers).
- [x] All plots are contour plots with outliers or pseudocolor plots.
- [x] A numerical value for number of cells or percentage (with statistics) is provided.

Methodology

Sample preparation: Cells were treated with experimental drugs and incubated for 48 hours. Following the incubation the cells were harvested using trypsin and fixed in ice cold 70% ethanol. To define the different cell cycle phases, cells were stained with Propidium iodide. To prevent intercalation with RNA, the fixed cells were treaed with RNaseA

Instrument: LSR FORTESS A was used to run the samples

Software: FCS express was used to analyze the data

Cell population abundance: In all the experiments 10,000 cellular events were recorded

Gating strategy: We gated the singlet population for analysis

- [ ] Tick this box to confirm that a figure exemplifying the gating strategy is provided in the Supplementary Information.

Magnetic resonance imaging

Experimental design

Design type

Design specifications

Behavioral performance measures

Imaging type(s)

Field strength

Sequence & imaging parameters

Area of acquisition

Diffusion MRI ☐ Used ☐ Not used

Preprocessing

Preprocessing software

Normalization

Normalization template

Noise and artifact removal

Volume censoring

Statistical modeling & inference

Model type and settings

Effect(s) tested

Specify type of analysis: ☐ Whole brain ☐ ROI-based ☐ Both

Statistic type for inference

(See Eklund et al. 2016)

Correction

Models & analysis

n/a	Involved in the study
☐	☐ Functional and/or effective connectivity
☐	☐ Graph analysis
☐	☐ Multivariate modeling or predictive analysis

Functional and/or effective connectivity

Graph analysis

Multivariate modeling and predictive analysis

This checklist template is licensed under a Creative Commons Attribution 4.0 International License, which permits use, sharing, adaptation, distribution and reproduction in any medium or format, as long as you give appropriate credit to the original author(s) and the source, provide a link to the Creative Commons license, and indicate if changes were made. The images or other third party material in this article are included in the article's Creative Commons license, unless indicated otherwise in a credit line to the material. If material is not included in the article's Creative Commons license and your intended use is not permitted by statutory regulation or exceeds the permitted use, you will need to obtain permission directly from the copyright holder. To view a copy of this license, visit http://creativecommons.org/licenses/by/4.0/
