# Supplementary material for: Discrete vulnerability to pharmacological CDK2 inhibition is governed by heterogeneity of the cancer cell cycle
Source: Nat Commun. 2025 Feb 9;16:1476. doi: 10.1038/s41467-025-56674-4 (PMC11808123; doi:10.1038/s41467-025-56674-4)
Supplement: Supplementary file 3 — Description of Additional Supplementary Files [file 41467_2025_56674_MOESM3_ESM.pdf]

**Supplementary Data 1**: Differential gene expression analysis on MB157-WT, MCF7, HCC1806 and MB157-RB-del cells following the treatment with INX-315 (100 nM) up to 48 H. The analysis was done based on n=3 biological replicates. The statistical analysis was determined based on Wald test in the R package "edgeR".

**Supplementary Data 2**: DrugZ analysis from CRISPR screen in MiaPaCa-2 (n=1 biological replicate) and HCC1806 (n=2 biological replicates) in the absence and presence of INX-315 (500 nM). The p value was determined from n=4 different guide sequences for each gene using the statistical method in the DrugZ algorithm.

**Supplementary Data 3**: DrugZ analysis from CRISPR screen in MiaPaCa-2 (n=1 biological replicate) and HCC1806 (n=1 biological replicate) in the absence and presence of palbociclib (500 nM for HCC1806 and 250 nM for MiaPaCa-2). The p value was determined from n=4 different guide sequences for each gene using the statistical method in the DrugZ algorithm.
