# Supplementary material for: Discrete vulnerability to pharmacological CDK2 inhibition is governed by heterogeneity of the cancer cell cycle
Source: Nat Commun. 2025 Feb 9;16:1476. doi: 10.1038/s41467-025-56674-4 (PMC11808123; doi:10.1038/s41467-025-56674-4)
Supplement: Supplementary file 8 — Source data file [file 41467_2025_56674_MOESM8_ESM.zip › Source data file revised/Fig 1.pptx]

## Slide 1
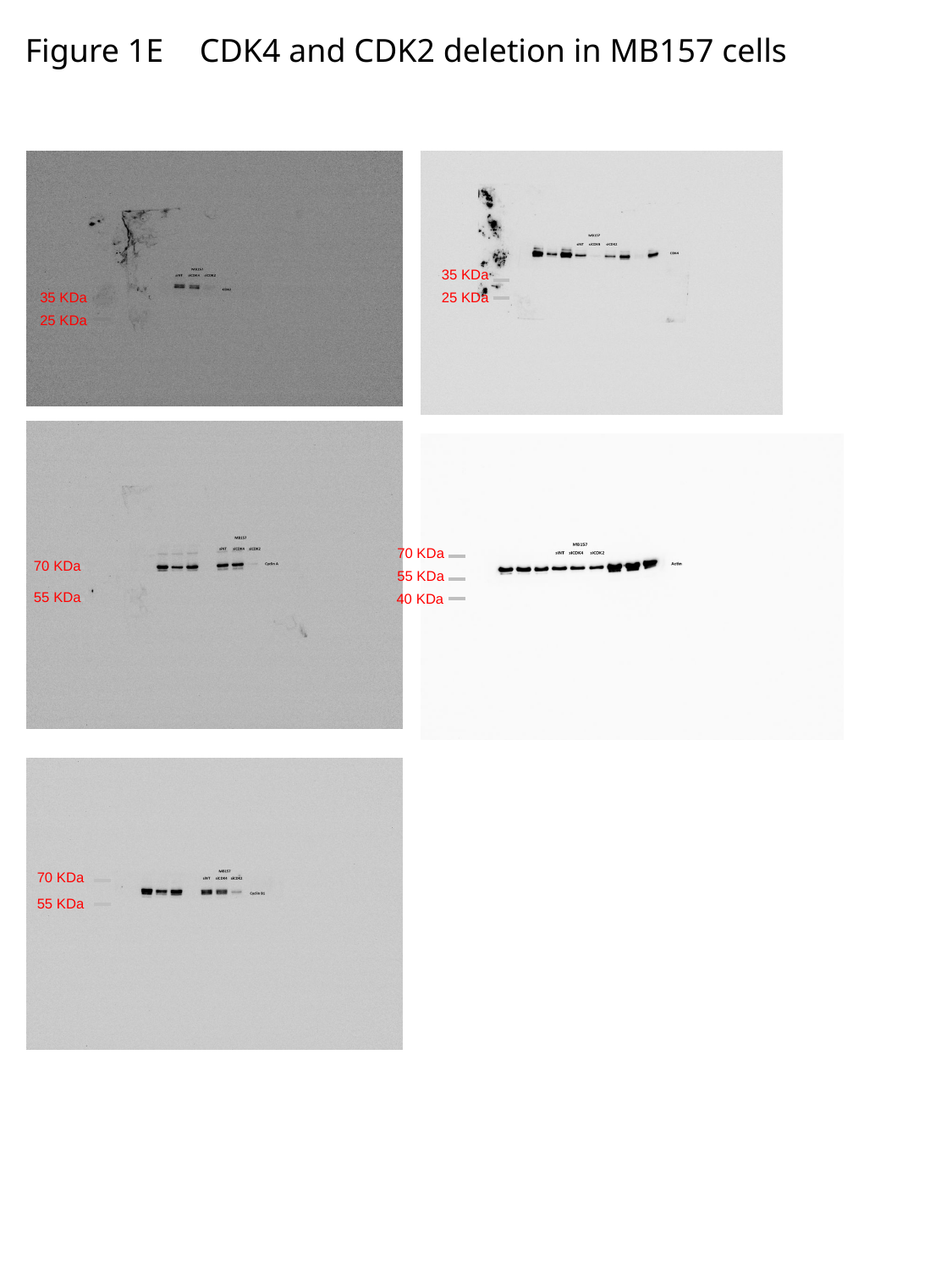

Figure 1E
CDK4 and CDK2 deletion in MB157 cells
35 KDa
25 KDa
35 KDa
25 KDa
70 KDa
70 KDa
55 KDa
55 KDa
40 KDa
70 KDa
55 KDa

## Slide 2
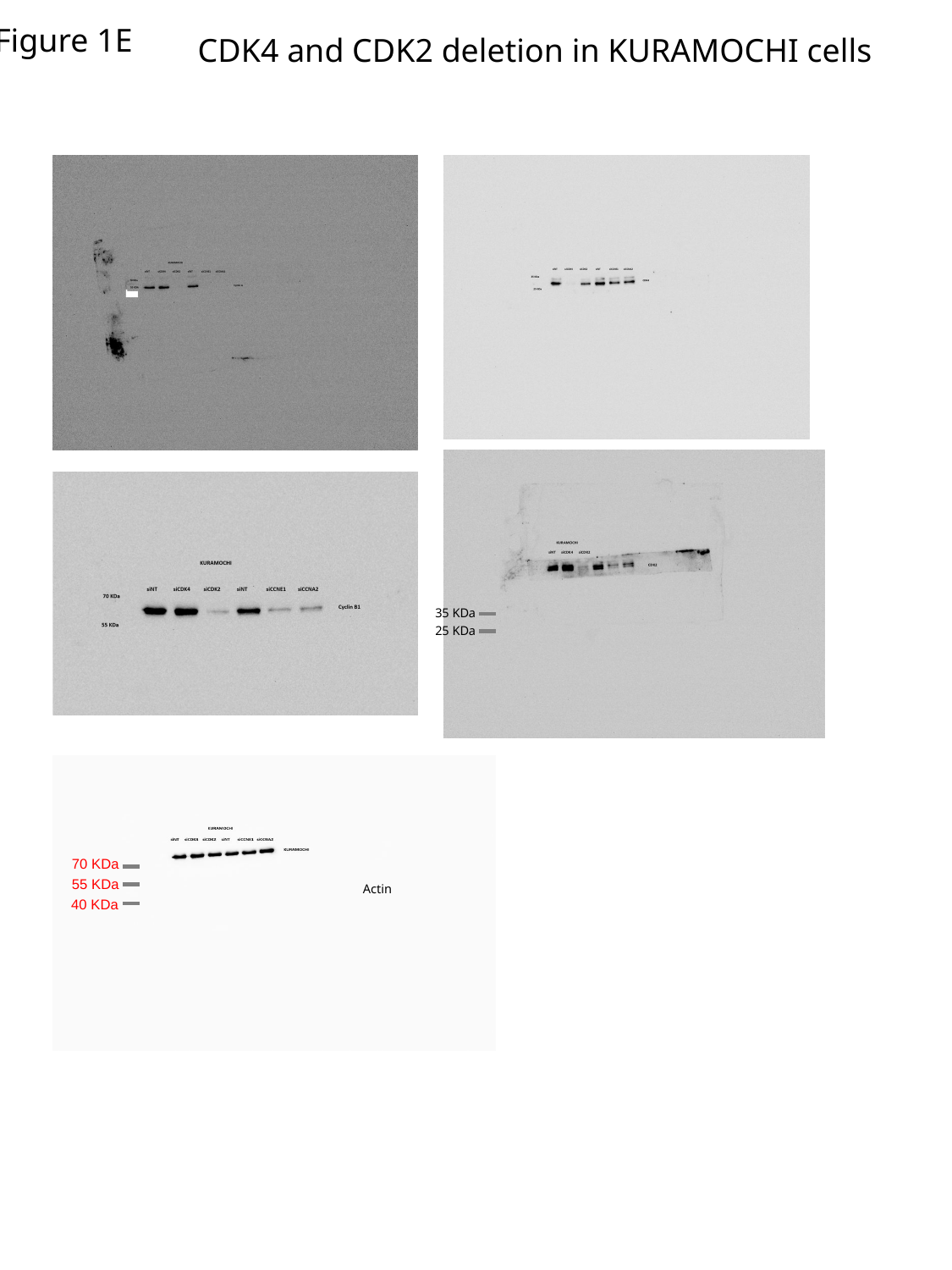

Figure 1E
CDK4 and CDK2 deletion in KURAMOCHI cells
35 KDa
25 KDa
70 KDa
55 KDa
Actin
40 KDa

## Slide 3
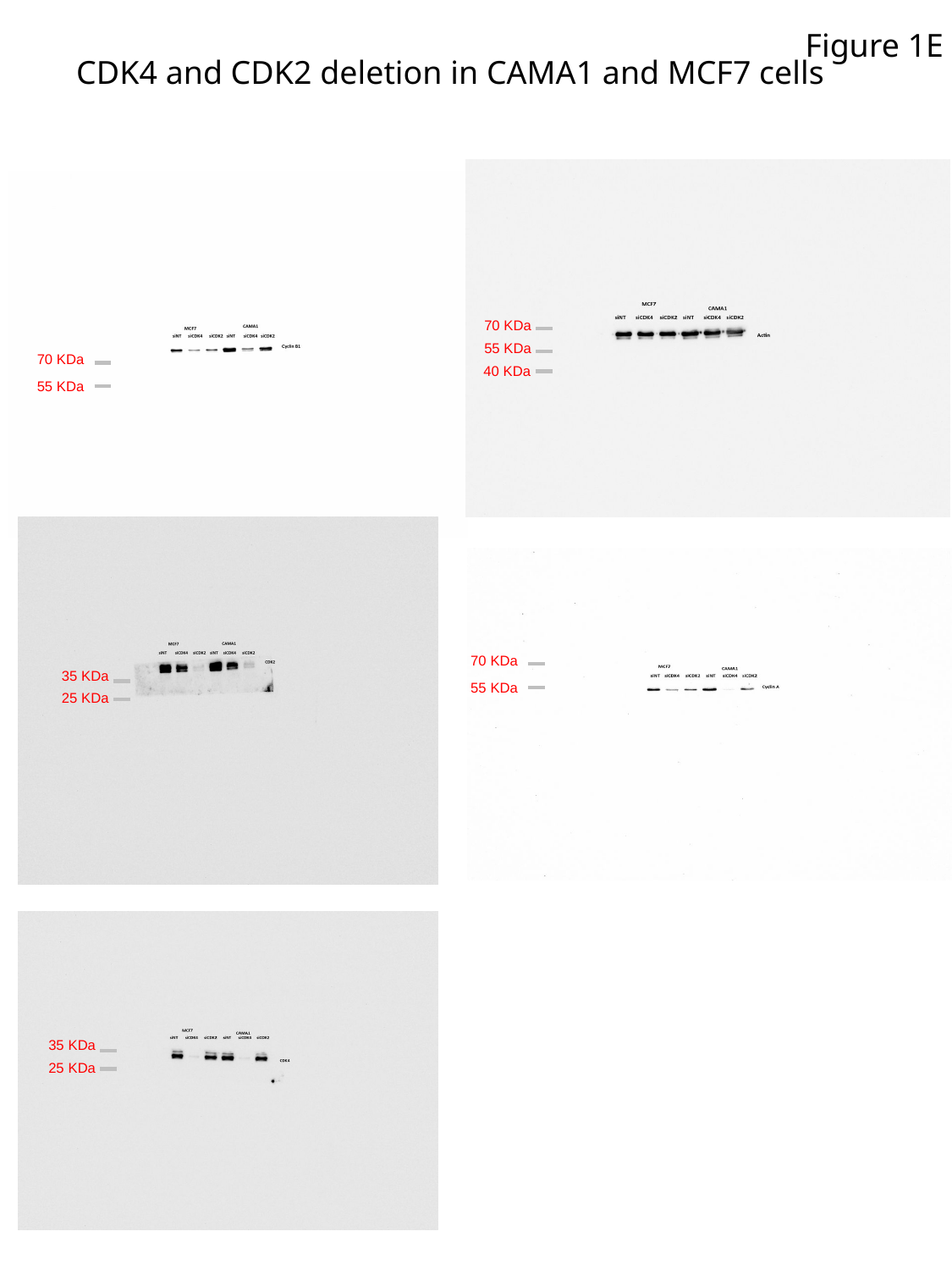

Figure 1E
CDK4 and CDK2 deletion in CAMA1 and MCF7 cells
70 KDa
55 KDa
70 KDa
40 KDa
55 KDa
70 KDa
35 KDa
55 KDa
25 KDa
35 KDa
25 KDa

## Slide 4
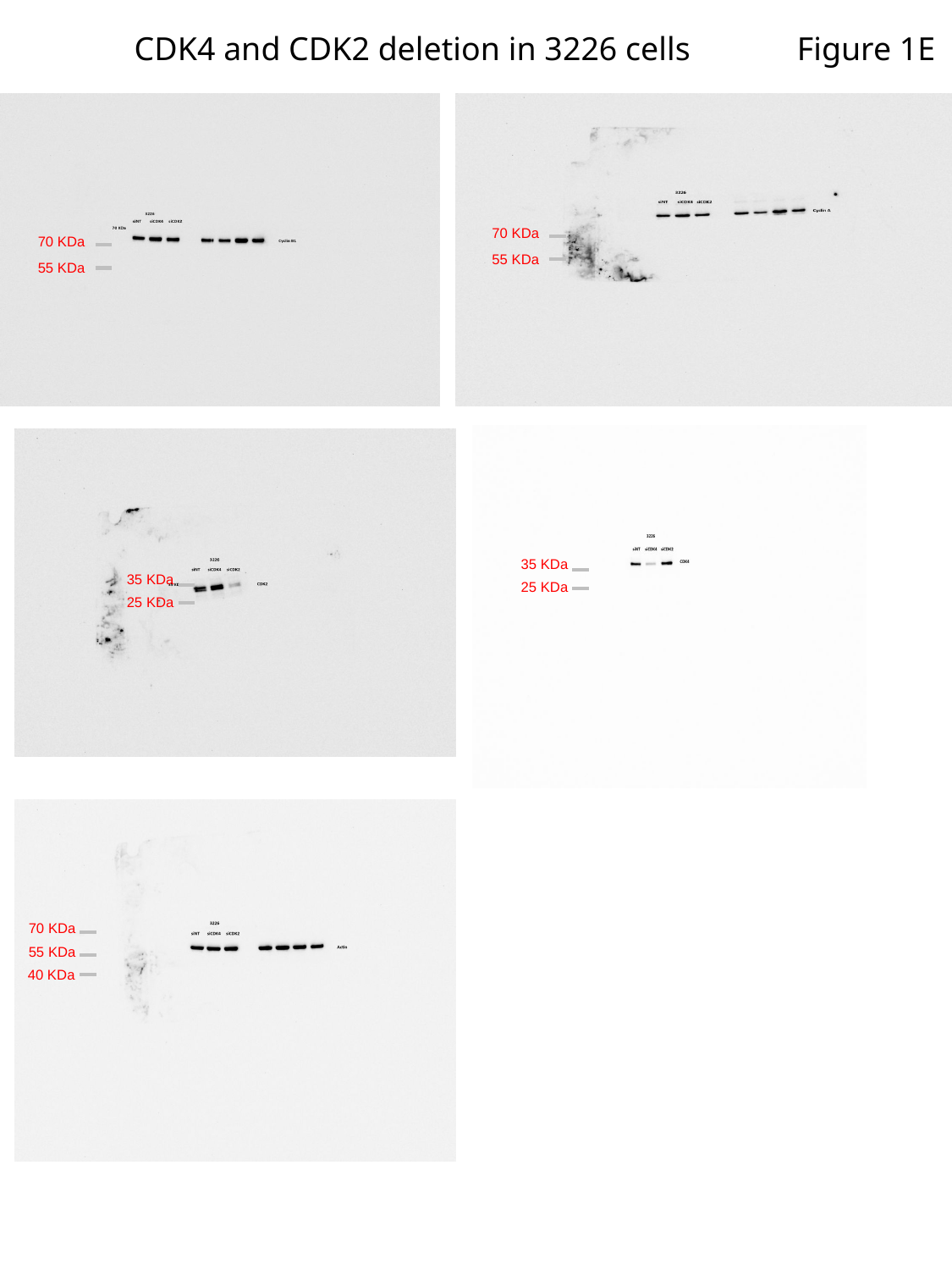

CDK4 and CDK2 deletion in 3226 cells
Figure 1E
70 KDa
70 KDa
55 KDa
55 KDa
35 KDa
35 KDa
25 KDa
25 KDa
70 KDa
55 KDa
40 KDa

## Slide 5
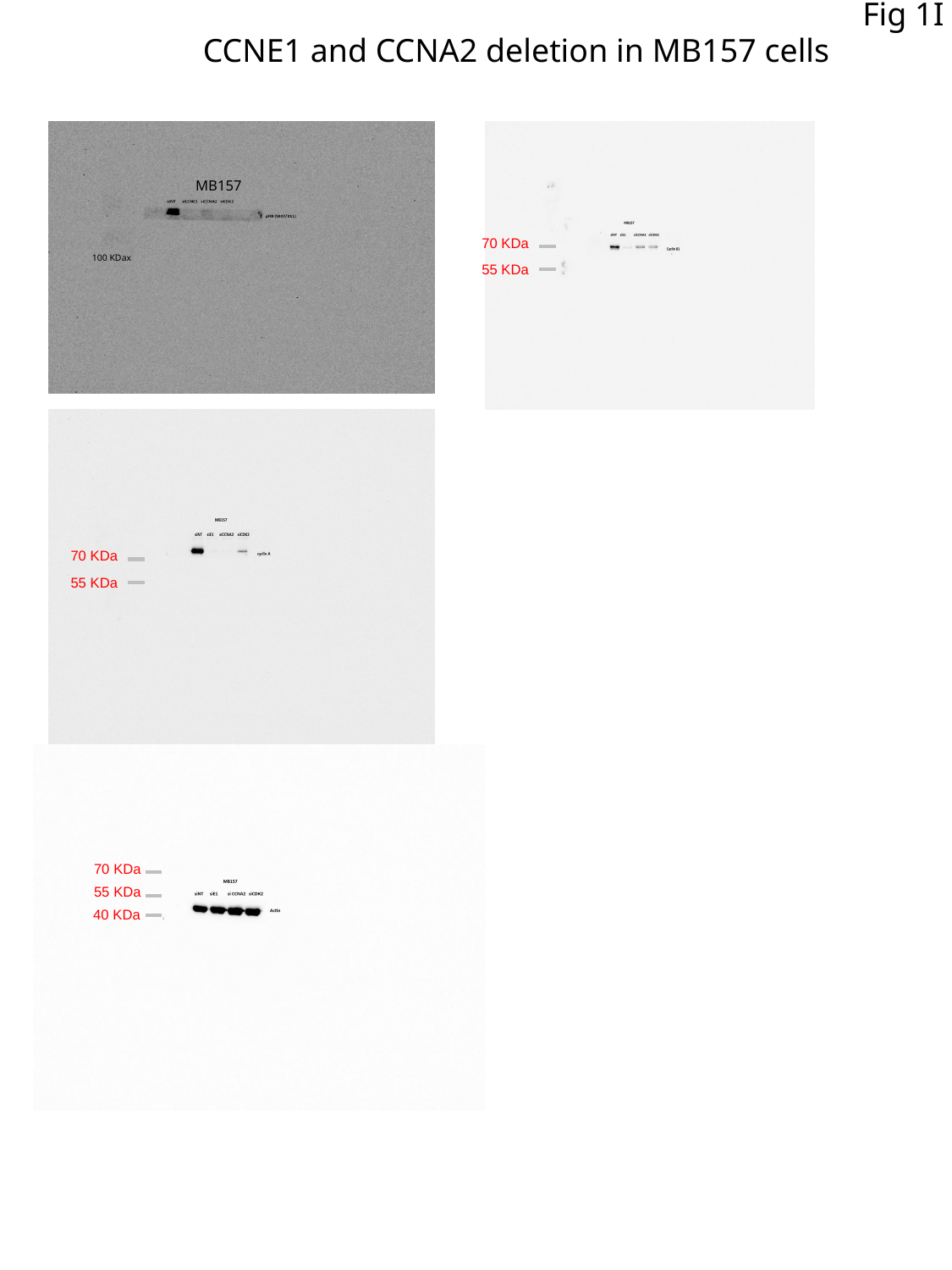

Fig 1I
CCNE1 and CCNA2 deletion in MB157 cells
MB157
70 KDa
100 KDax
55 KDa
70 KDa
55 KDa
70 KDa
55 KDa
40 KDa

## Slide 6
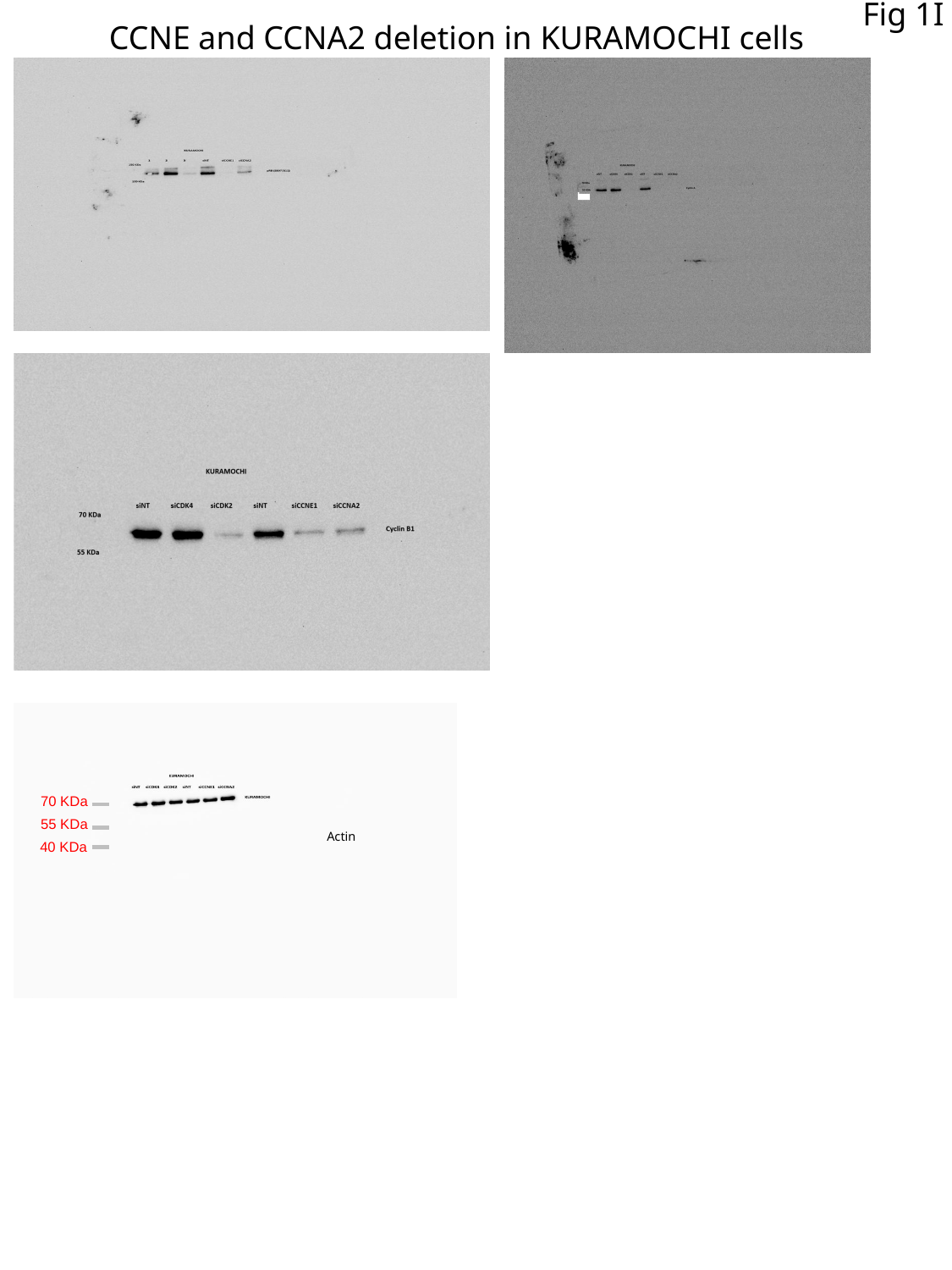

Fig 1I
CCNE and CCNA2 deletion in KURAMOCHI cells
70 KDa
55 KDa
Actin
40 KDa

## Slide 7
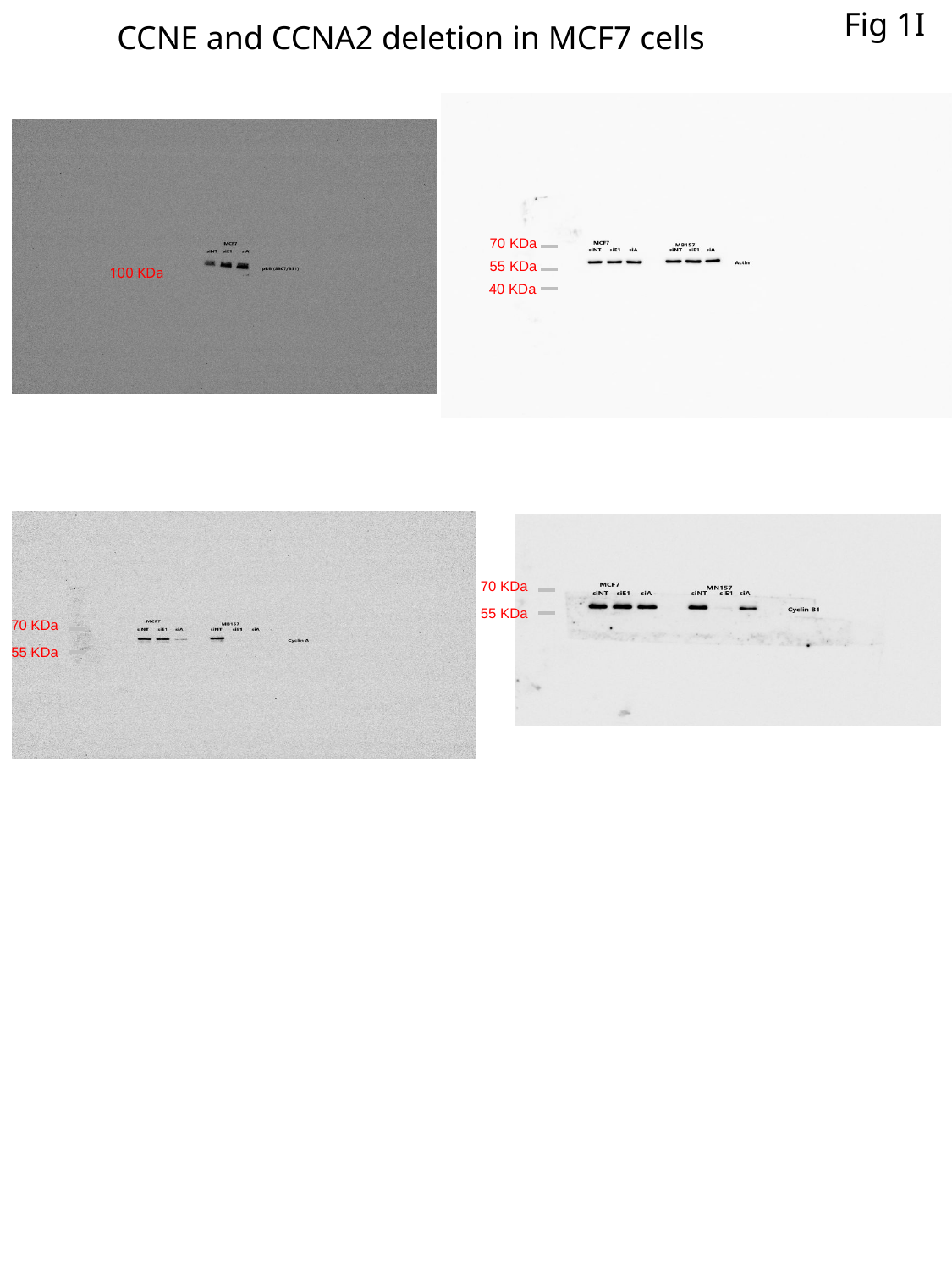

Fig 1I
CCNE and CCNA2 deletion in MCF7 cells
70 KDa
55 KDa
100 KDa
40 KDa
70 KDa
55 KDa
70 KDa
55 KDa
