# Supplementary material for: Discrete vulnerability to pharmacological CDK2 inhibition is governed by heterogeneity of the cancer cell cycle
Source: Nat Commun. 2025 Feb 9;16:1476. doi: 10.1038/s41467-025-56674-4 (PMC11808123; doi:10.1038/s41467-025-56674-4)
Supplement: Supplementary file 8 — Source data file [file 41467_2025_56674_MOESM8_ESM.zip › Source data file revised/Fig 3.pptx]

## Slide 1
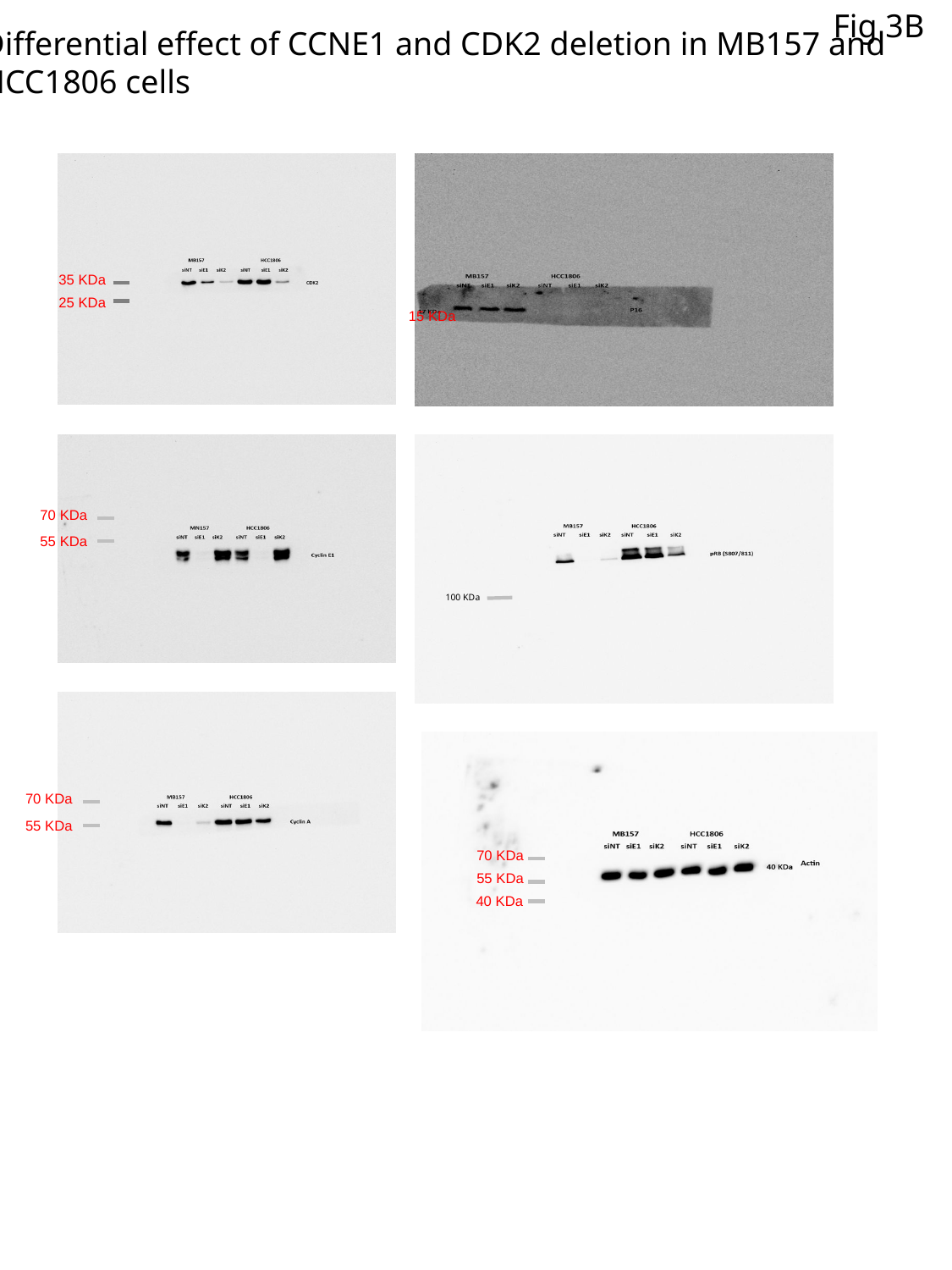

Fig 3B
Differential effect of CCNE1 and CDK2 deletion in MB157 and
HCC1806 cells
35 KDa
25 KDa
15 KDa
70 KDa
55 KDa
100 KDa
70 KDa
55 KDa
70 KDa
55 KDa
40 KDa

## Slide 2
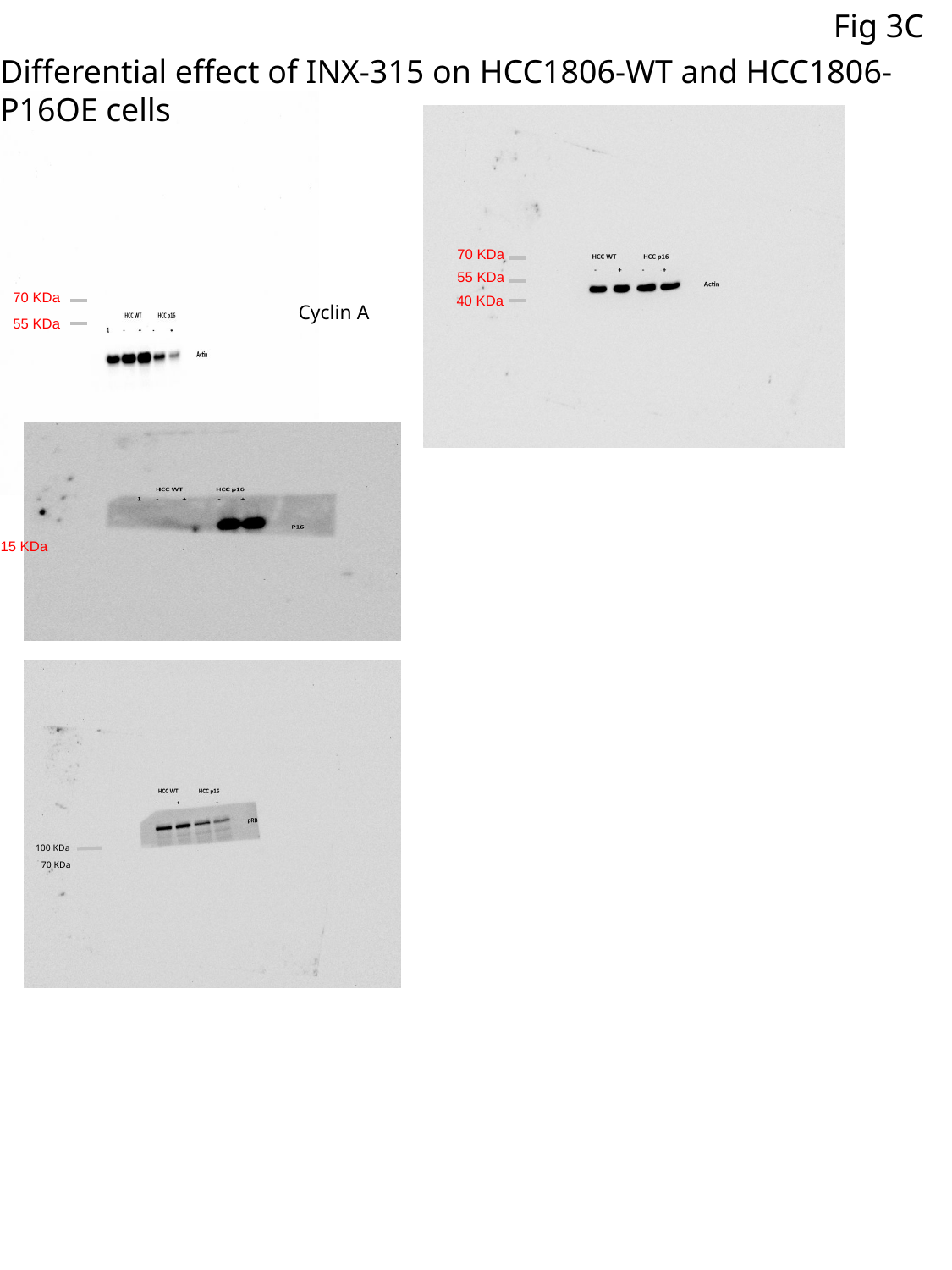

Fig 3C
Differential effect of INX-315 on HCC1806-WT and HCC1806-P16OE cells
70 KDa
55 KDa
70 KDa
40 KDa
Cyclin A
55 KDa
15 KDa
100 KDa
70 KDa

## Slide 3
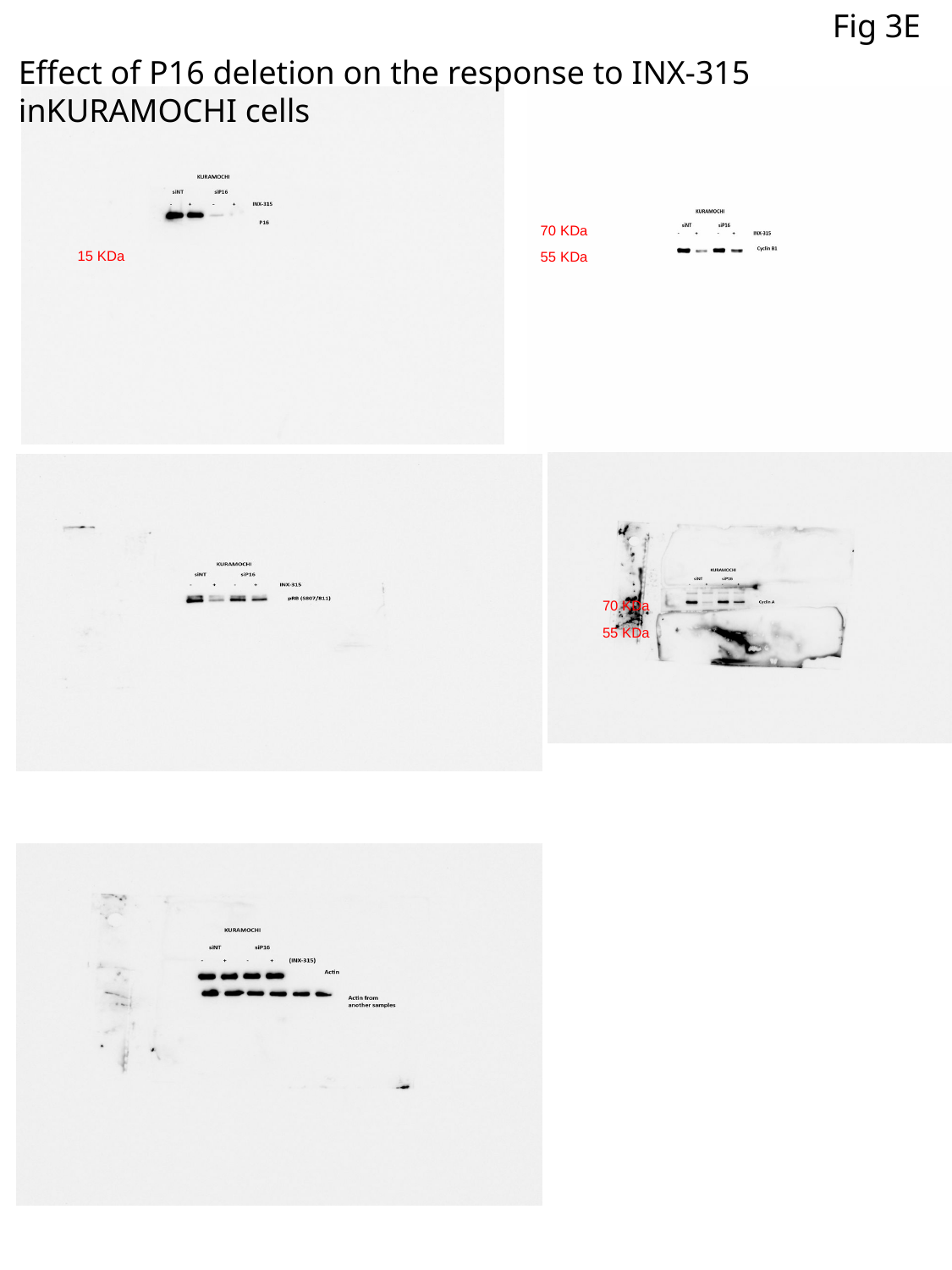

Fig 3E
Effect of P16 deletion on the response to INX-315 inKURAMOCHI cells
70 KDa
15 KDa
55 KDa
70 KDa
55 KDa

## Slide 4
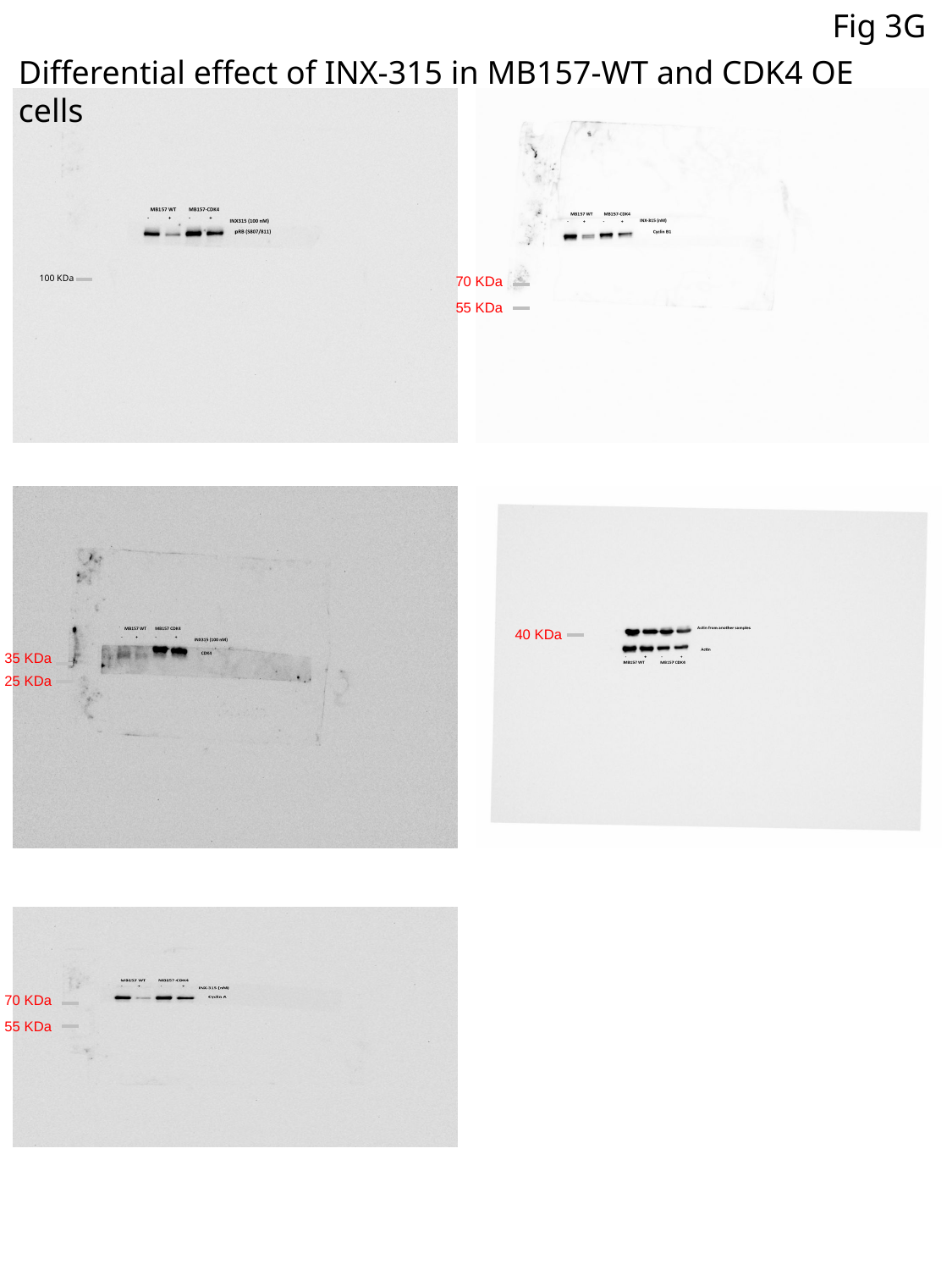

Fig 3G
Differential effect of INX-315 in MB157-WT and CDK4 OE cells
100 KDa
70 KDa
55 KDa
40 KDa
35 KDa
25 KDa
70 KDa
55 KDa
