# Supplementary material for: Discrete vulnerability to pharmacological CDK2 inhibition is governed by heterogeneity of the cancer cell cycle
Source: Nat Commun. 2025 Feb 9;16:1476. doi: 10.1038/s41467-025-56674-4 (PMC11808123; doi:10.1038/s41467-025-56674-4)
Supplement: Supplementary file 8 — Source data file [file 41467_2025_56674_MOESM8_ESM.zip › Source data file revised/Fig 6.pptx]

## Slide 1
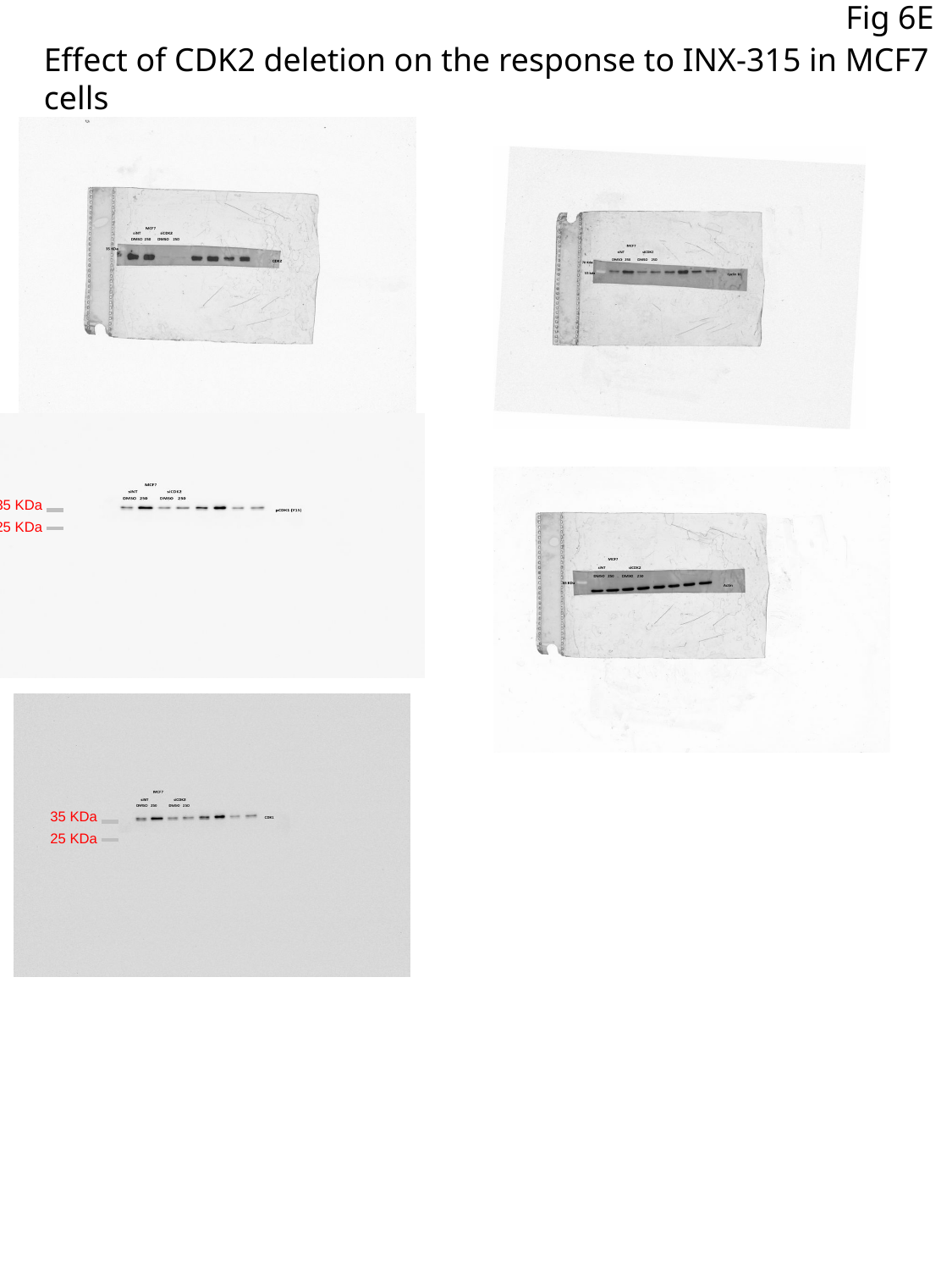

Fig 6E
Effect of CDK2 deletion on the response to INX-315 in MCF7 cells
35 KDa
25 KDa
35 KDa
25 KDa

## Slide 2
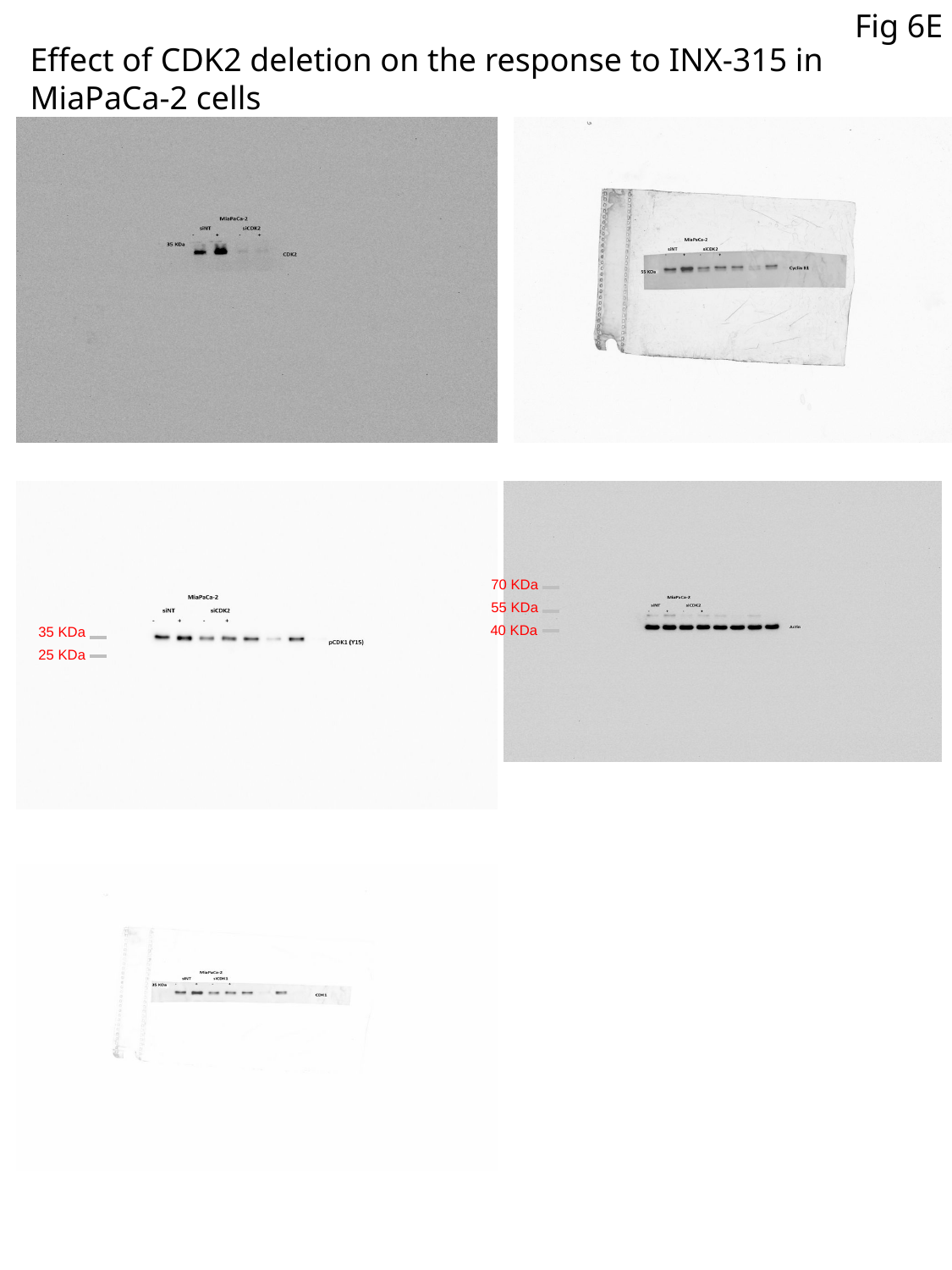

Fig 6E
Effect of CDK2 deletion on the response to INX-315 in MiaPaCa-2 cells
70 KDa
55 KDa
40 KDa
35 KDa
25 KDa
