# Supplementary material for: Discrete vulnerability to pharmacological CDK2 inhibition is governed by heterogeneity of the cancer cell cycle
Source: Nat Commun. 2025 Feb 9;16:1476. doi: 10.1038/s41467-025-56674-4 (PMC11808123; doi:10.1038/s41467-025-56674-4)
Supplement: Supplementary file 8 — Source data file [file 41467_2025_56674_MOESM8_ESM.zip › Source data file revised/Fig 8.pptx]

## Slide 1
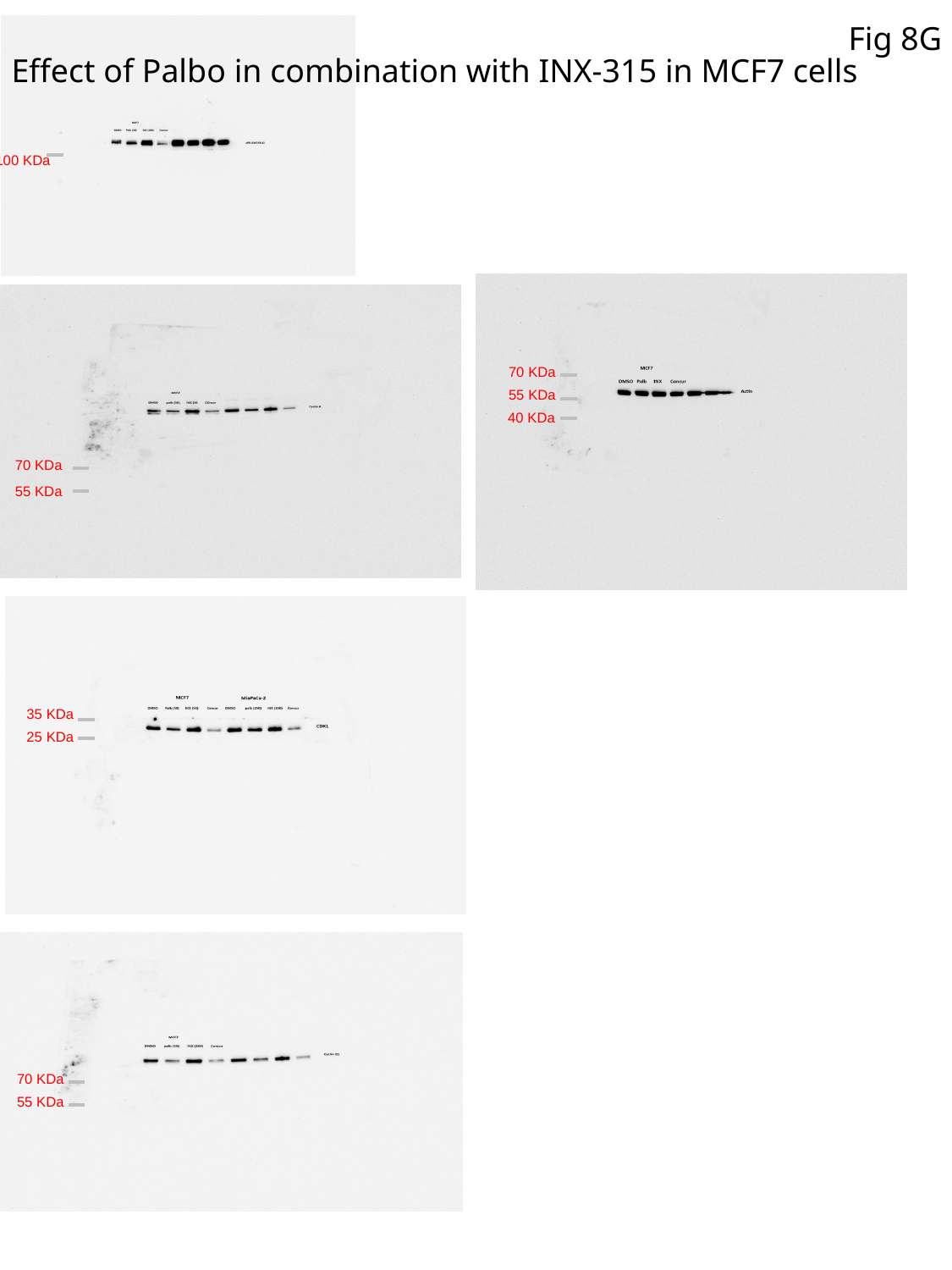

Fig 8G
Effect of Palbo in combination with INX-315 in MCF7 cells
100 KDa
70 KDa
55 KDa
40 KDa
70 KDa
55 KDa
35 KDa
25 KDa
70 KDa
55 KDa

## Slide 2
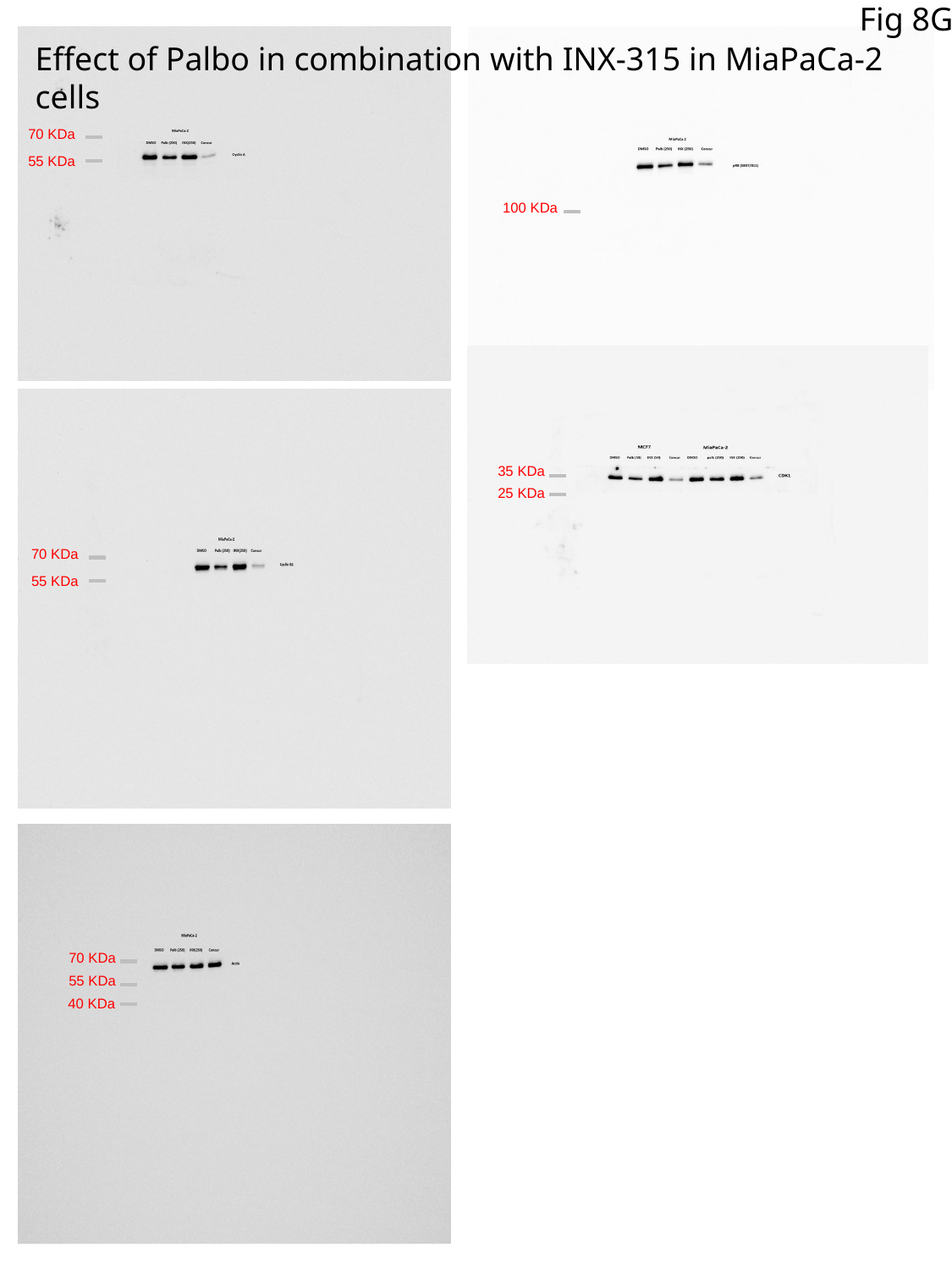

Fig 8G
Effect of Palbo in combination with INX-315 in MiaPaCa-2 cells
70 KDa
55 KDa
100 KDa
35 KDa
25 KDa
70 KDa
55 KDa
70 KDa
55 KDa
40 KDa

## Slide 3
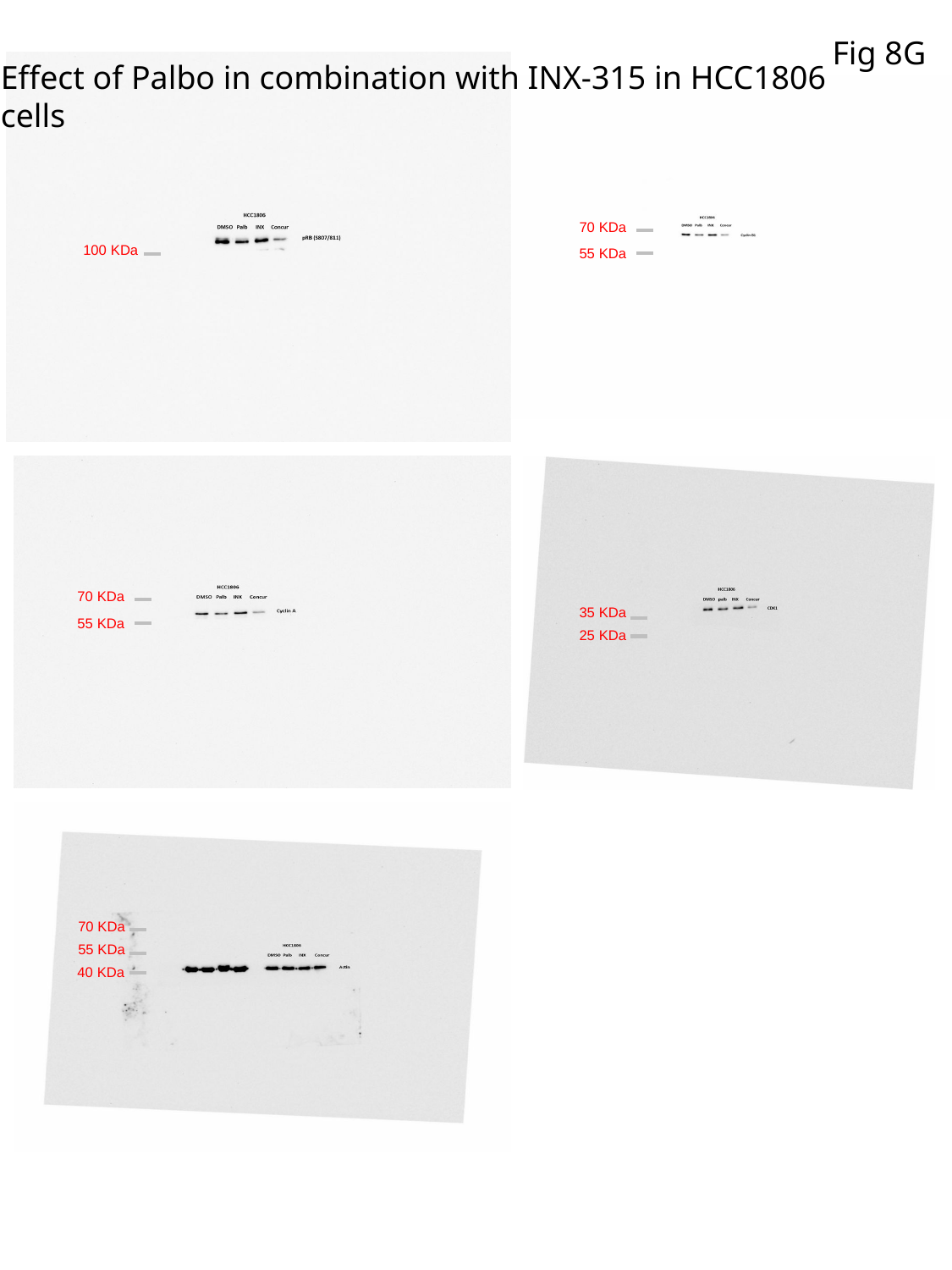

Fig 8G
Effect of Palbo in combination with INX-315 in HCC1806 cells
70 KDa
100 KDa
55 KDa
70 KDa
35 KDa
55 KDa
25 KDa
70 KDa
55 KDa
40 KDa

## Slide 4
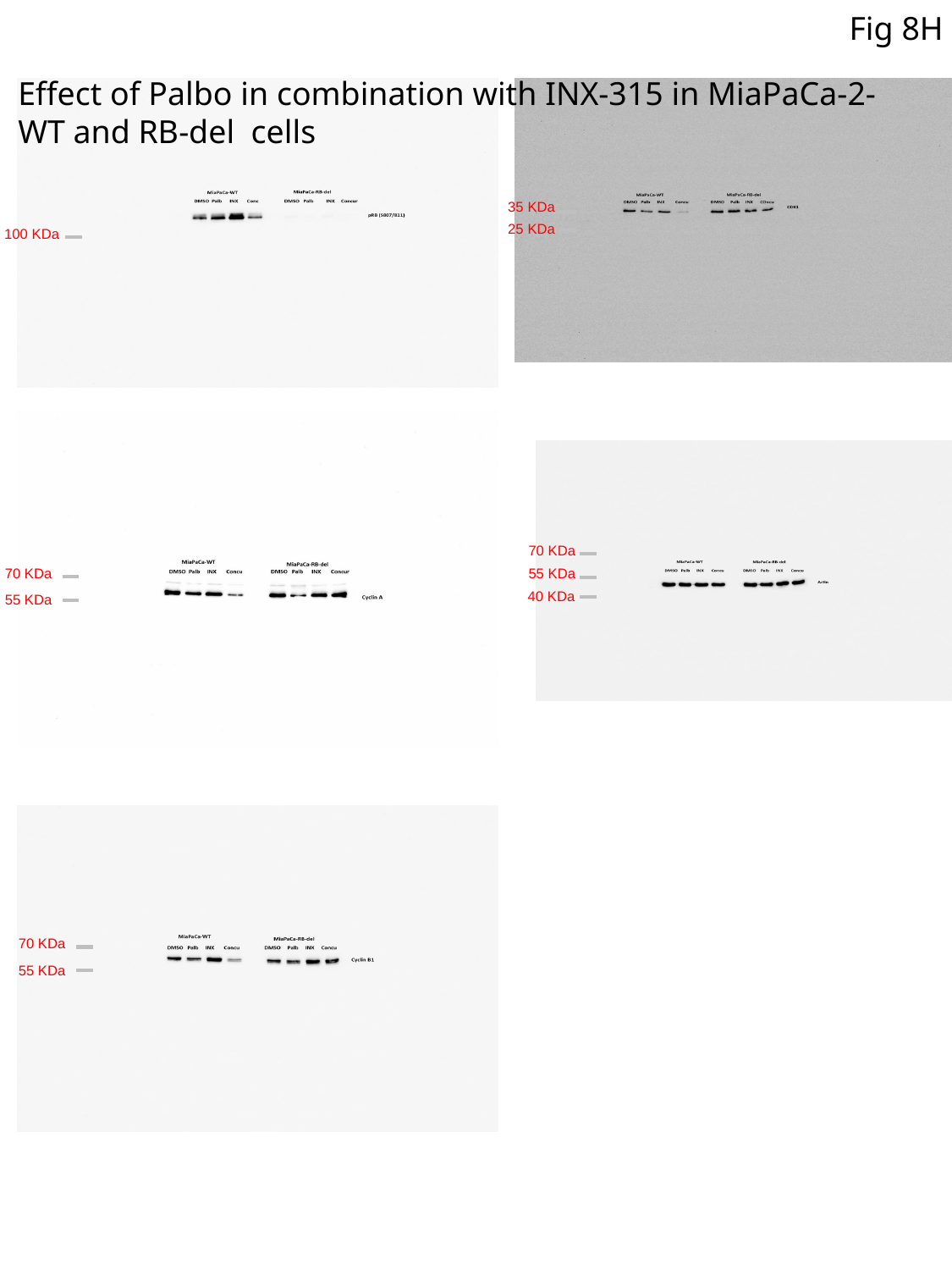

Fig 8H
Effect of Palbo in combination with INX-315 in MiaPaCa-2-WT and RB-del cells
35 KDa
25 KDa
100 KDa
70 KDa
70 KDa
55 KDa
40 KDa
55 KDa
70 KDa
55 KDa
