# Supplementary figures and images for: Discrete vulnerability to pharmacological CDK2 inhibition is governed by heterogeneity of the cancer cell cycle
Source: Nat Commun. 2025 Feb 9;16:1476. doi: 10.1038/s41467-025-56674-4 (PMC11808123; doi:10.1038/s41467-025-56674-4)

## Slide 1
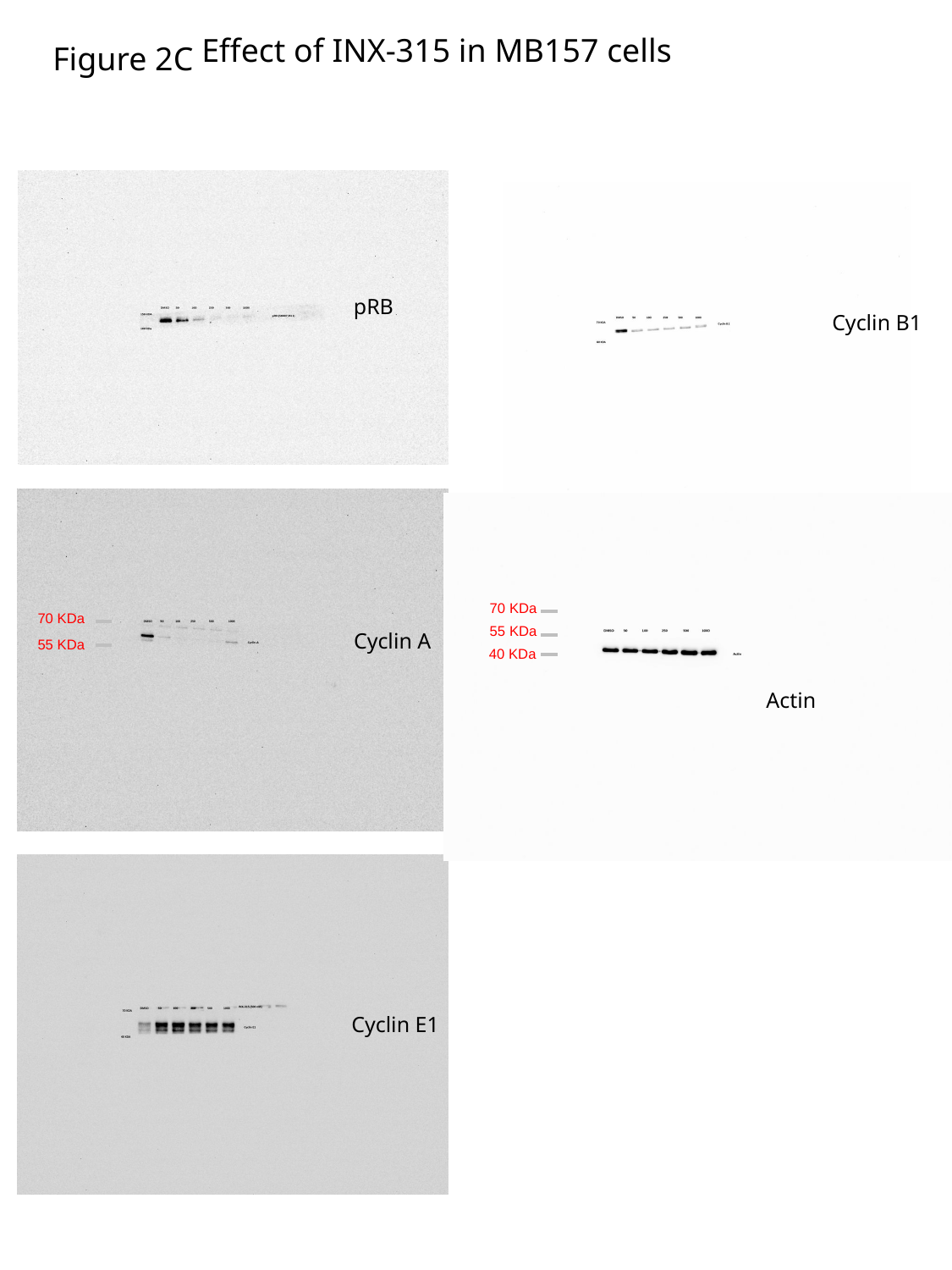

Effect of INX-315 in MB157 cells
Figure 2C
pRB
Cyclin B1
70 KDa
70 KDa
55 KDa
Cyclin A
55 KDa
40 KDa
Actin
Cyclin E1

Supplement: Supplementary file 8 — Source data file [file 41467_2025_56674_MOESM8_ESM.zip › Source data file revised/Fig 2C.pptx]
